# Water-Mediated Ion Selectivity in 2D MXene Channels

**DOI:** 10.1021/jacs.6c07978

**Published:** 2026-07-01

**Authors:** Yuan Zhang, Ming Chen, Teng Zhang, Tetiana Parker, Danzhen Zhang, Ruocun Wang, Hyunho Kim, Paweł Piotr Michałowski, Alexei Kornyshev, Yury Gogotsi

**Affiliations:** † Department of Materials Science and Engineering, 6527 Drexel University, Philadelphia, Pennsylvania 19104, United States; ‡ Department of Chemistry, Faculty of Natural Science, Molecular Sciences Research Hub, 4615 Imperial College London, White City Campus, Wood Lane, London W12 0BZ, U.K.; § Łukasiewicz Research Network, Institute of Microelectronics and Photonics, 01-919 Warsaw, Poland

## Abstract

At the Ångström scale, water confined between two-dimensional layers behaves fundamentally differently from bulk water; this behavior governs ion transport in natural and engineered nanofluidic systems, yet the mechanisms by which confined water mediates selective ion transport remain poorly understood. As classical descriptions of aqueous ion transport break down under extreme confinement, experimental studies often face challenges in controlling both nanoconfined structure and surface chemistry, limiting our ability to explain and predict water and ion behavior in subnanometer channels and membranes. Here, we show that 2D Ti_3_C_2_T*_x_* MXene nanosheets with precisely controlled interlayer spacing (0.9–5.0 Å), surface terminations, and electrode potentials provide a platform to systematically tune ion transport. Combined experimental measurements, including ion permeation, spatial secondary-ion mass spectrometry, and Fourier transform infrared spectroscopy, together with molecular dynamics simulations, reveal that ultranarrow confinement reorganizes confined water, imposes ion-specific energetic penalties for dehydration, and modulates ion–MXene interactions. Li^+^ permeation in horizontally aligned Ti_3_C_2_T*_x_* channels is 2 orders of magnitude faster than in conventional vertically aligned MXene membranes, while electrochemical surface charge modulation further regulates ion selectivity. These coupled effects of confinement, surface chemistry, and water-mediated energetics define a transport regime beyond classical diffusion, offering design principles for artificial ion channels and high-performance membranes for ion separation, water desalination, and sustainable water treatment technologies.

## Graphical abstract

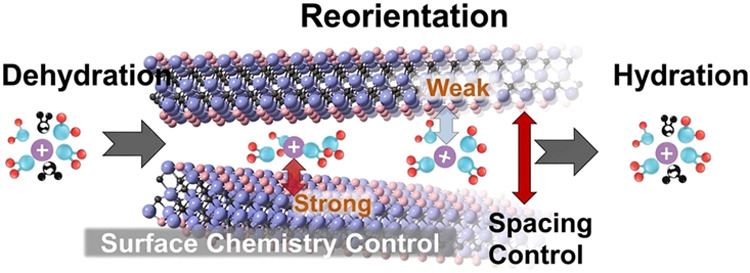

## Introduction

When ions move through subnanometer-scale confined spaces, their behavior differs significantly from bulk diffusion due to spatial constraints,^1,2^ surface interactions,^3,4^ and dehydration effects.^5–8^ Understanding ion transport mechanisms in 2D nanoconfinement is crucial for designing advanced materials and optimizing technologies across supercapacitors,^9–12^ blue energy harvesting,^13–15^ water purification,^1,16,17^ lithium extraction,^5^ sensing,^18^ memristors,^19^ catalysis,^20^ and bioinspired ion and mass transport.^21^

2D materials, such as graphene oxide (GO),^22,23^ transition metal dichalcogenides (TMDs),^24^ phyllosilicate-based clays,^25–27^ and MXenes,^28–31^ serve as platforms for subnanometer 2D ion channels due to their tunable, uniform interlayer spacing under good flake alignment.^32^ These materials create Ångström- to nanometer-scale channels that restrict ion transport, particularly influencing ion selectivity, mobility, and interactions. Among these 2D materials, MXene channels allow precise subnanometer confinement down to ∼1–3 Å, approaching and below the bare ionic diameter of ions. This extreme confinement enables strong control over water–ion coupling, which is critical for ion transport. At subnanometer confinement, water undergoes profound structural and dielectric changes: the out-of-plane dielectric constant is strongly suppressed,^33^ while the in-plane effective dielectric constant of water layer in the gap is enhanced^34^ relative to bulk water. This confinement-induced dielectric anisotropy directly alters ion hydration and ion–surface interactions under electrical fields, fundamentally redefining transport mechanisms in 2D channels. Moreover, MXene offers high tunability of lattice composition and surface termination chemistry that can switch the surface charge and hydrophilicity/hydrophobicity.^35^ Its high electrical conductivity further allows electrochemical modulation of surface charge, enabling dynamic control over ion transport.^36^

Prior studies have explored tuning interlayer spacing via ion/solvent intercalation, van der Waals assembly, or pillaring,^22,23,37–40^ revealing that narrow channels favor smaller or weakly hydrated ions while suppressing larger or strongly hydrated species.^7,40,41^ Different distances of ions from the channel walls in 2D confinement can lead to distinct ionic mobilities, even for ions with similar hydrated sizes.^42^ Confinement-induced difference in cation and anion mobilities reflects key electrostatic and surface-interaction effects.^41,43^

Collectively, these studies unravel multifarious aspects of confined ion transport and, in particular, establish the interlayer spacing as a key control parameter. However, spacing modulation is inherently coupled with structural deformation, surface charge redistribution, and water reorganization, particularly under extreme subnanometer confinement (<5 Å), thereby preventing independent control of hydration shells and ion–surface interactions. Consequently, this complicates the understanding of how the confined water structure, surface chemistry, and electrostatics collectively govern ion mobility.

Here, we establish a Ti_3_C_2_T*_x_* MXene nanochannel platform with fixed interlayer spacings from 0.9 to 5.0 Å and a Ti_3_C_2_Cl_2_ nanochannel terminated with Cl, providing a distinct interfacial environment for confined water while eliminating swelling induced by ion hydration. Molecular dynamics (MD) simulations and electrochemical modulation reveal how confined water, surface chemistry, and electrode potential jointly regulate diffusion and desorption. This platform provides systematic design principles for subnanometer ion channels and high-performance membranes, enabling controlled ion transport for water desalination, selective ion separation, ion recovery, and other ionotronic devices.

## Results and Discussion

### MXene Channel Design

To systematically investigate ion transport under tunable nanoconfinement, we constructed a series of MXene-based ion channels with precisely controlled interlayer spacing (described in the Experimental Section in the Supporting Information). The interlayer spacing of the Ti_3_C_2_T*_x_* MXene channel was modulated via intercalant exchange or hydration with different species (as described in the Supporting Information), including protons (labeled as H–Ti_3_C_2_T_*x*_), water (H_2_O–Ti_3_C_2_T_*x*_), and tetramethylammonium, TMA^+^ (TMA-Ti_3_C_2_T_*x*_). In addition, a Li^+^-intercalated MXene film obtained after LiCl-assisted delamination and spray coating was used and denoted as Li–Ti_3_C_2_T_*x*_. Delaminated Li–Ti_3_C_2_Cl_2_ single-layer MXene was synthesized via the molten-salt method and delaminated, as described in our previous work.^44^ The obtained MXene films were subsequently dried and physically constrained (see the Experimental Section and Supporting Figures S1–S3) with a fixed structure and *d*-spacing, which is defined as the periodic distance between equivalent atomic planes of adjacent MXene sheets (Figure 1a).

**Figure 1:**
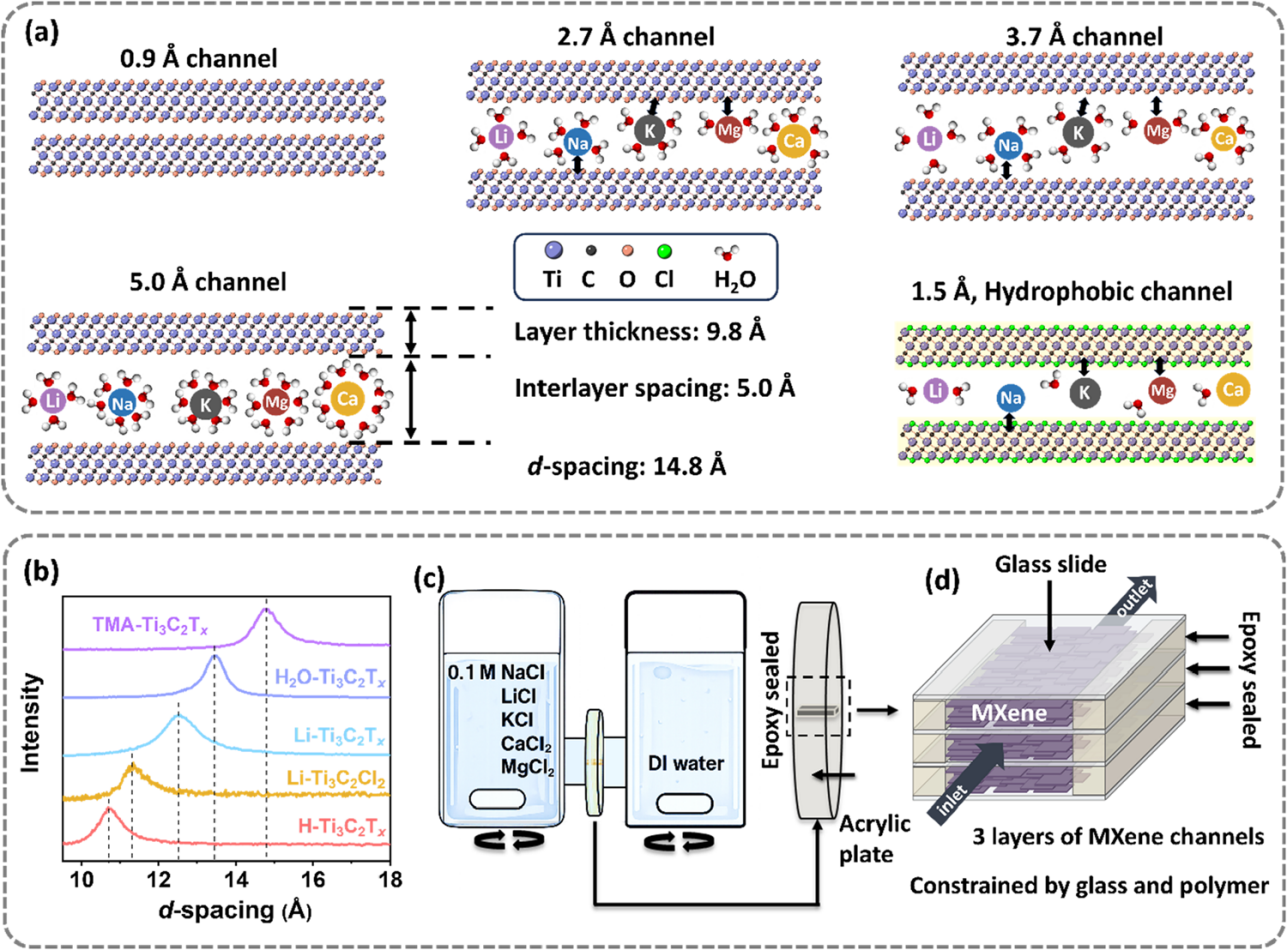
Design of MXene channels with controlled interlayer spacing and surface chemistry. (a) Schematic of MXene confinements with different interlayer spacing and surface chemistry; (b) XRD characterization of MXenes with varying *d*-spacing; (c) cell setup for ion permeation measurement, where ion transport is driven by a concentration gradient between two reservoirs and the solution concentrations evolve during permeation; and (d) schematic of constrained MXenes channels with controlled *d*-spacing.

The *d*-spacing was determined by X-ray diffraction (XRD) measurements (Figure 1a,b), spanning a range of 10.7 Å to 14.8 Å. Considering the slab thickness of a monolayer has been determined by STEM and density functional theory (DFT) as 9.0–10 Å, depending on surface terminations,^45,46^ according to the reported data, no significant difference in layer thickness between Ti_3_C_2_T*_x_* and Ti_3_C_2_Cl_2_ is expected. Therefore, a monolayer thickness of 9.8 Å was used to estimate the effective interlayer confinement throughout this work. Specifically, the interlayer spacing of the MXene channels follows the order of H–Ti_3_C_2_T_*x*_ (0.9 Å) < Li–Ti_3_C_2_Cl_2_ (1.5 Å) < Li–Ti_3_C_2_T_*x*_ (2.7 Å) < H_2_O–Ti_3_C_2_T_*x*_ (3.7 Å) < TMA-Ti_3_C_2_T_*x*_ (5.0 Å), demonstrating systematic and reproducible control over the confinement dimension. Importantly, MXene channels were physically constrained between glass slides to prevent expansion and changes in interlayer spacing during the experiment, forcing the MXene flakes to align horizontally and form well-aligned nanochannels. Under this geometry, ion transport predominantly occurs along the in-plane direction within the nanoconfinement, in contrast to free-standing MXene membranes with flakes oriented normal to the ion flow direction.^36,40,47^ The in-plane configuration substantially shortens the diffusion length and reduces ion transport tortuosity, providing a more efficient and controllable platform for probing ion selectivity arising from confined MXene channels, compared with out-of-plane transport in stacked MXene membranes.

Ion permeation and selectivity were measured in a concentration-driven ion transport configuration (Figure 1c,d). The MXene ion channel was horizontally positioned and sealed within a transparent acrylic plate (see the Experimental Section and Supporting Information, Figure S1). The plate was placed between two reservoirs: a feed solution (a mixture of 0.1 M NaCl, LiCl, KCl, CaCl_2_, and MgCl_2_) and a permeate solution (initially Milli-Q water). Driven solely by the concentration gradient, ions were transported through the MXene nanochannels from the feed side to the permeate side. After 48 h, the permeate water was collected and analyzed by ion chromatography.

### Cation Selectivity under Steric and Surface Chemistry Effects

Figure 2a presents the permeation rates of various cations through different MXene membranes. The relevant ion parameters governing transport under nanoconfinement, including bare ion size, hydrated diameter, and hydration energy, are listed in Supporting Table S1.^48–50^ The Atomic Li:Ti ratios of Li–Ti_3_C_2_T_*x*_ and Li–Ti_3_C_2_Cl_2_ are measured using inductively coupled plasma optical emission spectroscopy (ICP-OES) measurement and are listed in Supporting Table S2. When H–Ti_3_C_2_T*_x_* was used as the ion channel, the average interlayer spacing was ∼0.9 Å. Under such extreme confinement, ion transport encounters the highest impedance and necessarily involves dehydration. As a result, all cations exhibit low permeation rates (30–40 mol·m^–2^ h^–1^, Figure 2a) as ion transport is dominated by nonselective interflake gaps and edge pathways rather than well-defined nanochannels, leading to negligible ion selectivity. In contrast, when a pristine Li^+^ delaminated MXene dry film (Li–Ti_3_C_2_T_*x*_) with an average interlayer spacing of around 2.7 Å was employed, a much more pronounced ion separation with distinct cation permeation rates was observed. Among all cations, Li^+^ and Ca^2+^ exhibit the highest permeability and selectivity, whereas Na^+^, K^+^, and Mg^2+^ show substantially lower permeation rates. Divalent cations, such as Mg^2+^ and Ca^2+^, taken at the same feed-side molarity as the monovalent ions, experience a larger chemical potential difference across the channel; only Ca^2+^ demonstrates enhanced permeation, while Mg^2+^ remains strongly hindered, suggesting that ion transport under confinement is not governed by concentration gradients or charge alone. Instead, dehydration and confinement-induced energetic penalties play a dominant role. Among monovalent cations, where dehydration energies are significantly lower than those of divalent ions, selective permeation follows the trend of bare ionic size. Li^+^ shows the highest permeation rate compared to other cations; its permeation rate measured in a single salt system under different temperatures is shown in Supporting Figure S5a.

**Figure 2:**
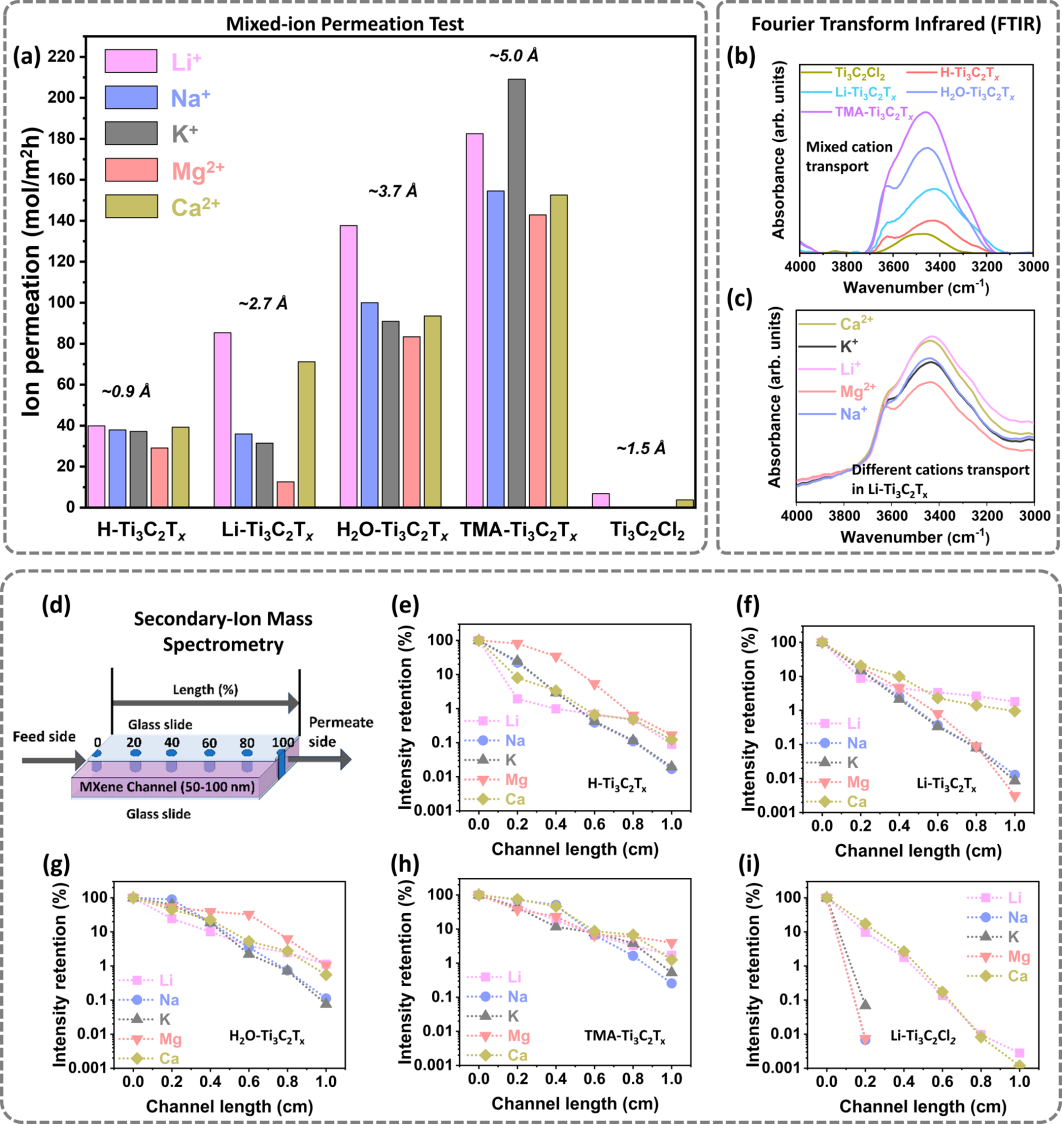
Ion selectivity of constrained MXene channels with different interlayer spacings and surface terminations. (a) Ion permeation rate from a mixture of different cations (0.1 M) through MXene channels to deionized water. (b, c) Confined water is characterized by Fourier transform infrared (FTIR) spectroscopy under different confinement environments. (b) With mixed cation (0.1 M LiCl, NaCl, KCl, MgCl_2_, CaCl_2_ aqueous solution) under different interlayer spacings, pillared by intercalants. (c) With different single cations in the Li–Ti_3_C_2_T_*x*_ channel. (d) Schematic illustration of secondary-ion mass spectrometry (SIMS) characterization on a constrained MXene ion channel at different lengths. (e–i) Elemental intensity retention at different lengths within H–Ti_3_C_2_T_*x*_, Li–Ti_3_C_2_T_*x*_, H_2_O–Ti_3_C_2_T_*x*_, TMA-Ti_3_C_2_T_*x*_, and Li–Ti_3_C_2_Cl_2_ channels.

To examine the effect of relaxed confinement, the Ti_3_C_2_T_*x*_ channel was first hydrated with more confined water (H_2_O–Ti_3_C_2_T_*x*_, ∼3.7 Å), increasing interlayer spacing and enhancing overall cation permeation. While all cations diffuse faster in the wider channels, ionic selectivity weakens. Further expansion to ∼5.0 Å via TMA^+^ pillaring, approaching hydrated ion sizes, greatly increases permeation rates. In this regime, Li^+^-dominant selectivity observed in Li–Ti_3_C_2_T_*x*_ disappears, and K^+^, with hydrated diameter best matching the channel, exhibits the highest permeation, whereas other cations follow similar trends as in Li–Ti_3_C_2_T_*x*_. Overall, weak confinement with preserved hydration favors fast transport but reduces selectivity.

When Li–Ti_3_C_2_Cl_2_ was applied as the ion channel, the effectively hydrophobic Cl-terminated surface significantly impeded ion transport. Under these conditions, the permeation of Na^+^, K^+^, and Mg^2+^ falls below the detection limit. The permeation of Li^+^ and Ca^2+^ was still detectable, but exhibited much lower rates (<10 mol·m^–2^ h^–1^). Li–Ti_3_C_2_Cl_2_ exhibits a high ion selectivity with slow ion permeation. This behavior indicates that the ion diffusion rate is not only related to the space size and hydration energy of ions with different valence numbers but also to the amount of water transported along with the ions. Consequently, the surface hydrophilicity/hydrophobicity of the MXene plays a critical role in regulating ion diffusivity and selectivity.

While many reported 2D membranes have shown an ion permeation rate below 1 mol·m^–2^ h^–1,^ ^6,36,40,47^ , Li–Ti_3_C_2_T_*x*_ exhibits a Li^+^ ion permeation rate of around 90 mol·m^–2^ h^–1^ (nearly 2 orders of magnitude higher), and the Li^+^/Mg^2+^ selectivity is calculated in Supporting Figure S6. Li–Ti_3_C_2_Cl_2_ exhibits exceptionally high Li^+^ selectivity, with the concentrations of Na^+^, K^+^, and Mg^2+^ below the detection limit. The constrained channel architecture shows structural integrity after 14 days of immersion in the feed solution, as evidenced in Supporting Figure S5b.

All in all, our results show that the ion selectivity and permeation rate can be controlled directly via the confinement and chemistry of the channels. The horizontally aligned channels allow much lower tortuosity for ion transport, sufficiently shortening the ion diffusion and allowing for the ion selectivity.

### Fourier Transform Infrared (FTIR) Spectroscopy of Confined Water

To directly probe the role of confined water in regulating ion transport within MXene nanochannels, Fourier transform infrared (FTIR) spectroscopy in attenuated total reflection (ATR) mode was used to characterize the state and amount of confined water under different channel configurations (see Experimental Section in the Supporting Information). As shown in Figure 2b, in mixed solutions, the FTIR spectra of MXene channels with different interlayer spacing exhibit two characteristic features in the water O–H stretching region (3100–3900 cm^–1^). In mixed-cation solutions, spectra exhibit a weak shoulder at 3630 cm^–1^ from dangling −OH terminations and a broader 3300–3600 cm^–1^ band corresponding to water in solvation shells. After ion transport experiments, the overall absorbance intensities of confined water increase with interlayer spacing, indicating a larger amount of water accommodated in wider channels. Notably, for Li–Ti_3_C_2_Cl_2_ channels, the spectral feature around 3630 cm^–1^ is absent, in agreement with its hydrophobic surface −Cl terminations that suppress interfacial water and disrupt intermolecular hydrogen bonding. For water molecules associated with ion solvation shells, a gradual shift toward stronger O–H vibration is observed as interlayer spacing increases. This reflects a weakening of cation-H_2_O interactions due to the increased number of solvating water molecules around each ion. The strong cation–water interaction directly weakens the force constant of the covalent O–H bond, resulting in a red shift of the O–H stretching vibration.^51^ With increasing interlayer spacing, the enhanced hydration environment facilitates the cotransport of water molecules with cations, thereby accelerating ion transport. In contrast, for Li–Ti_3_C_2_Cl_2,_ the hydrophobic surface inhibits the cotransport of water molecules, leading to stronger cation–channel interactions and consequently much lower ion diffusivity and permeability.

To further understand the hydration ability of ions within the same MXene confinement, a single-cation (0.1 M for one kind of cation) permeation test was conducted in a constrained Li–Ti_3_C_2_T_*x*_ channel (Figure 2c). Among all cations, Li^+^ and Ca^2+^ exhibit the highest OH stretching absorbance, indicating a higher total number of confined water molecules. The hydration degree of the MXene channel under different permeation conditions follows the trend Li^+^ > Ca^2+^ > Na^+^ > K^+^ > Mg^2+^, consistent with the ion-selective permeation test in Figure 2a. The fingerprint region under different confinement environments, as shown in Supporting Figure S7­(a,b), reveals no pronounced shift in bending vibration frequencies of Ti–O surface termination. Different cation transports have little impact on the surface termination of Li–Ti_3_C_2_T_*x*_. X-ray photoelectron spectroscopy (XPS) reveals that the MXene channel remains chemically stable, with no oxidation observed after 2 years of storage in a vacuum desiccator and 14 days soaked in the feed solution, Supporting Figure S7­(c–f).

These results show that by controlling the amount of water in ion solvation shells via confinement and surface chemistry engineering, ion transport can be controlled to achieve a high selectivity.

### Ion-Selective Transport within MXene Confinements

To gain spatially resolved insight into ion transport kinetics within the MXene confinement, secondary-ion mass spectrometry (SIMS) characterization was employed (Figure 2d–i).^52^ After conducting 48 h ion permeation tests in a 2 cm long channel, the ion channels were collected for SIMS analysis. From the feed side to the permeate side along the ion permeation direction, the ion traces were measured at 4 mm intervals (20% of the channel total length) (Figure 2d). The measured cation intensity decay is proportional to the ion concentration decay within the MXene confinement (initial values are shown in Supporting Figure S8). Given the ultralong ion transport pathway, the ion distributions within the thin-film layers follow a nonsteady state. This will facilitate comparison of diffusion rates of ions across different MXene nanoconfinements.

For H–Ti_3_C_2_T_*x*_ (Figure 2e), all cations exhibit nonlinear intensity decay along the transport direction, indicating slow and diffusion-limited transport under extreme confinement. As the interlayer spacing was further increased to around 2.7 Å (Figure 2f), Li^+^ and Ca^2+^ started to show a nonlinear distribution under the log scale. The change in the slope as a function of transport distance indicates an ion distribution with faster ion diffusion relative to the other cations, and rearrangement of ion hydration shells as ion transport further to the permeate side. Small Li^+^ cations and larger divalent Ca^2+^ cations with higher hydration energy tend to have higher mobility, and this is a combined effect of size and hydration structure. As the interlayer further increased to around 3.7 Å in H_2_O–Ti_3_C_2_T_*x*_ (Figure 2g), less falling-off was observed; this is due to a higher ion transport diffusivity in the confinement, with the addition of one layer of confined water and increasing the interlayer spacing by 1 Å. In TMA-Ti_3_C_2_T_*x*_ (Figure 2h), the slope of intensity decay is further decreased, showing an enhanced ion permeability and diffusivity for all cations within the nanoconfinement. As hydrophobic Li–Ti_3_C_2_Cl_2_ is applied (Figure 2i), the cations transport within the MXene confinement shows a rapid decay with a boundary of ion distribution, and all cation intensities show linear distribution in the log scale with a constant slope. While Na^+^, K^+^, and Mg^2+^ became undetectable as the permeation length along the channel reached further than 0.2 cm, Li^+^ and Ca^2+^ showed very low intensities as ions reached the permeate side. Among all cations, Mg^2+^ shows a decreased slope in ion distribution along the transport pathway on a log scale, while Li^+^ and Ca^2+^ show an increased slope compared with all other cations.

Combining ion permeation, FTIR, and SIMS results, we conclude that ion transport permeability and diffusivity are strongly related to the degree of ion hydration within the nanoconfinement, whereas controlling the interlayer spacing can change the ion transport energy barrier and allow for selective ion transport. Tuning surface hydrophilicity and hydrophobicity can also alter ion diffusion from liquid-like to solid-like behavior.

### Effect of Confined Water

Ion transport in subnanometer MXene channels deviates fundamentally from classical diffusion because the simultaneous confinement of ions and water alters the hydration structure and local friction.^53,54^ From a microscopic perspective, ion motion through the slit can be conceptually decomposed into three sequential processes (Figure 3a): (i) ion intercalation accompanied by partial dehydration, (ii) hydration-shell restructuring and water reorientation within the confined region, and (iii) ion rehydration upon exiting the channel. To explicitly resolve how confined water and solvation energetics govern ion mobility and selectivity under these conditions, MD simulations were therefore employed (Supporting Figure S9), which showed the following.

**Figure 3:**
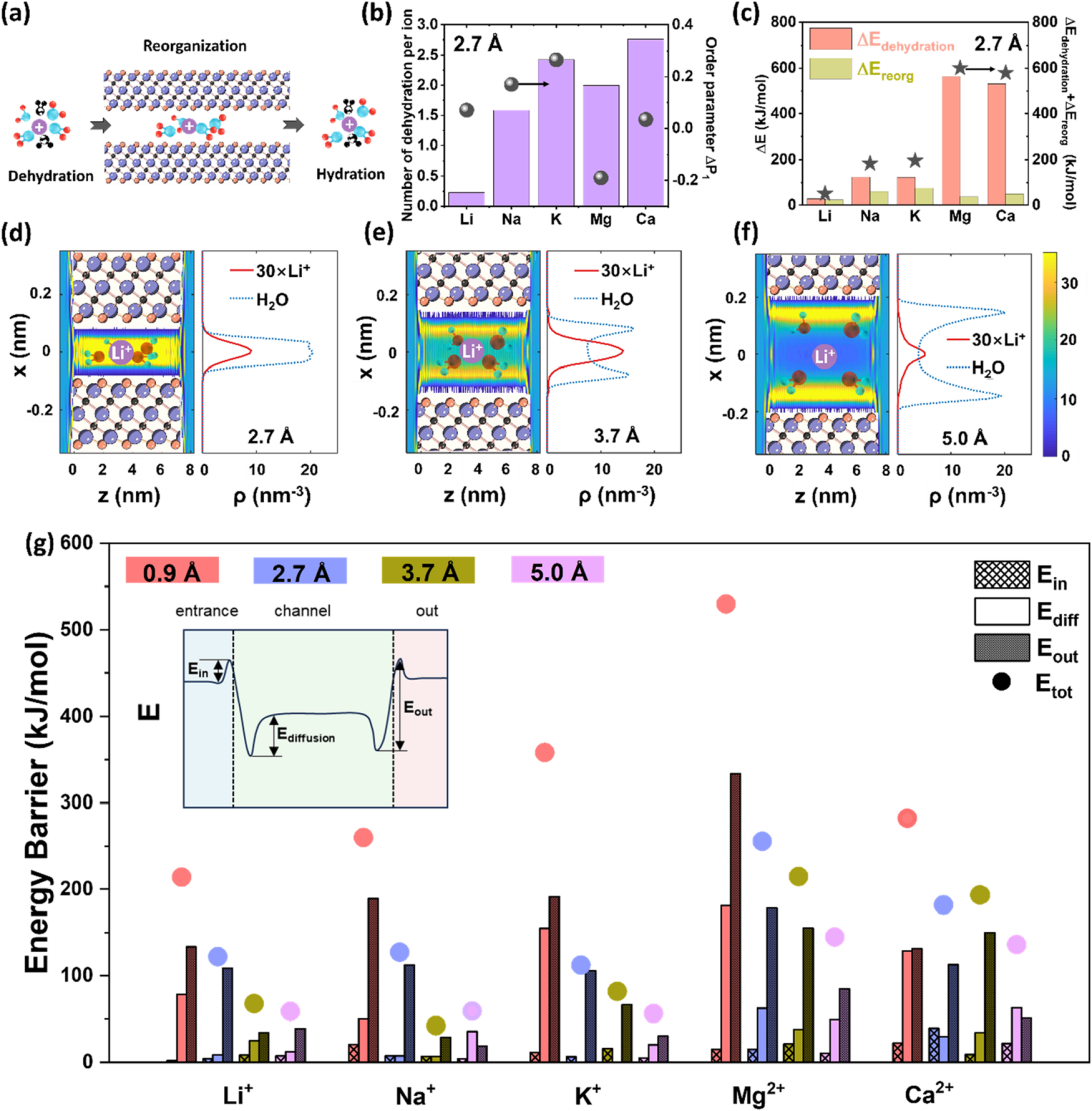
Water-mediated microscopic mechanism of selective ion transport in Subnanometer MXene nanochannels. (a) Schematic illustration of the coupled dehydration and water-reorganization processes during ion intercalation into the MXene nanochannel. (b) Evolution of hydration-shell structure and first-order orientational order parameter *P*_1_ for different cations upon entering the MXene slit. (c) Decomposition of the energetic penalties associated with ion dehydration (Δ*E*_dehydration_) and confined-water reorganization (Δ*E*_reorg_) for different cations. (d–f) Spatial distributions (ρ) of ions (Li^+^) and water molecules inside the MXene slit at different interlayer spacings 2.7 Å (d), 3.7 Å (e), and 5.0 Å (f). (g) Free-energy profiles associated with ion intercalation (*E*_in_), in-plane diffusion (*E*_diff_), pore exit (*E*_out_), and the sum of all three terms (*E*_tot_) at different interlayer spacings.

In bulk electrolytes, ions are fully hydrated by an isotropic solvation shell with a bulk-like water structure (Supporting Figures S10–S11). Upon entering the subnanometer MXene slit, the hydration environment becomes confined in an ion-dependent manner. At an interlayer spacing of 2.7 Å, Li^+^ retains a nearly intact solvation shell (Figure 3b), whereas other cations undergo pronounced dehydration, with Na^+^ losing ∼1.5 water molecules relative to its bulk coordination, for instance. Consistent trends are observed across the alkali series, following the sequence Li^+^ < Na^+^ < K^+^, while divalent cations display a similar hierarchy, Mg^2+^ < Ca^2+^. Beyond dehydration, nanoconfinement induces distortions of the remaining solvation structure, which can be quantified by the first-order orientational order parameter *P*_1_ (see the Supporting Information). All ions affect the orientational order of water molecules (Δ*P*_1_ = *P*_1_^pore^ – *P*_1_^bulk^) under confinement, reflecting hydration-shell restructuring under confinement (Figure 3b). Notably, Li^+^ shows the smallest rearrangement of its hydration shell, whereas the degree of hydration shell distortion increases with ionic size. Li^+^ provides the best hydration-shell fit in the case of narrow confinements, whereas larger hydrated ions experience progressively poorer geometric compatibility.

These ion-dependent structural responses directly translate into distinct energetic penalties associated with ion intercalation. As shown in Figure 3c, Li^+^ exhibits the lowest overall energetic cost among monovalent cations, with a combined dehydration and reorganization energy of approximately 20 kJ/mol, exhibiting an optimal hydration-shell fit. In contrast, Na^+^ and K^+^ incur substantially higher dehydration penalties on the order of ∼100 kJ/mol. Although their dehydration energies are comparable, Na^+^ displays a slightly lower reorganization energy than K^+^, indicating a less severe solvation-shell distortion. For divalent cations, the energetic landscape is dominated by strong electrostatic interactions: both Mg^2+^ and Ca^2+^ exhibit very large dehydration penalties approaching ∼500 kJ/mol, with Mg^2+^ being unfavorable due to its rigid hydration shell. These ion-dependent energetic penalties persist across the entire confinement range studied, although increasing interlayer spacing progressively reduces their magnitude (Supporting Figures S9–S11). These energetic penalties establish the thermodynamic foundation for the ion-specific transport behavior under confinement.

We then examined the spatial distributions of ions and water molecules within the MXene slit channel. In the most strongly confined H–Ti_3_C_2_T*_x_* channel, where the effective pore size is comparable to the bare ionic diameter, ion entry is associated with a prohibitively large free-energy barrier. As a result, Li^+^ ions rarely enter the slit within the accessible simulation time scale, and the corresponding density profiles are therefore not shown. As the interlayer spacing increases from 2.7 Å to 5.0 Å, Li^+^ remains preferentially located near the center of the slit, whereas water undergoes pronounced redistribution and reorientation (Figure 3d–f). At strong confinement (2.7 Å), water molecules adopt a quasi-planar arrangement (Figure 3d), indicative of a highly constrained hydration environment. With increasing spacing, water molecules progressively reorient, with hydrogen atoms pointing toward the negatively charged MXene surfaces (Figure 3e). Upon further expansion to 5.0 Å, water accumulates near the pore walls, leaving the central region populated primarily by Li^+^ with a reduced local water density (Figure 3f).

Similar confinement-induced redistribution and reorientation of water were observed for other cations (Supporting Information Figure S12–S18), although the extent of redistribution depends on ion-specific hydration strength. Collectively, these structural features provide a microscopic picture of how ion mobility is modulated under nanoconfinement. As water molecules accumulate near the pore walls with the increase of the interlayer spacing, the component of the effective dielectric constant of the confined water, normal to the slit plane, progressively increases, enhancing dielectric screening and thereby weakening the effective ion-surface coupling, particularly for ions residing near the center of the channel (Supporting Figure S14). Under such conditions, ions experience reduced resistance to in-plane motion, consistent with the experimentally observed enhancement of the ionic transport under moderate confinement. Meanwhile, the diminished differentiation of hydration environments among different ions under an enlarged confinement reduces ion-specific energetic contrasts. Consequently, these confinement-induced redistributions of ions and water are associated with enhanced ionic diffusivity, albeit at the expense of a reduced ion selectivity.

While the above analysis characterizes the static free-energy landscape, ion transport is ultimately governed by the dynamic process of entering, traversing, and exiting the slit pore. To quantify the free-energy barriers along this pathway, we computed the potential of mean force (PMF) for ion permeation. Figure 3g summarizes the free-energy barriers associated with ion intercalation into the slit (*E*_in_), in-plane diffusion within the confinement (*E*_diffusion_), and ion exit back into the bulk solution (*E*_out_) for cations at various spacings. Across all spacings, the intercalation barrier remains small and weakly ion-dependent, indicating that ion entry is not the rate-limiting step. In contrast, the diffusion and exit barriers are substantially larger and exhibit pronounced ion- and spacing-dependent variations, thereby dominating the overall free-energy cost of transport through the MXene slit. We further used a Smoluchowski-type bottleneck estimate,^55^ *k*_*i*_ ∝ *D*_*i*_^conf^exp (−Δ*G*_*i*_^‡^/*k*_B_ *T*), where Δ*G*_*i*_^‡^ is the dominant diffusion/exit barrier and *D*_*i*_^conf^ represents the confined diffusion prefactor, to compare the relative kinetic barriers of different ions.

Under strong confinement, Li^+^ consistently exhibits the lowest diffusion and exit barriers among all investigated cations, accounting for its higher permeability and preferential transport. By comparison, Na^+^ and K^+^ display substantially higher diffusion and exit barriers due to their larger size and more strongly distorted hydration structures under confinement, while divalent cations (Mg^2+^ and Ca^2+^) exhibit the highest barriers owing to strong electrostatic interactions and large dehydration penalties. As the interlayer spacing increases, the diffusion and exit barriers are progressively reduced for all cations, and the energetic separation between ions becomes less pronounced, indicating weakened ion-specific energetic discrimination and diminished selectivity. In the limit of severe confinement (0.9 Å), where dielectric screening by water is largely absent (since we see no water in such channel), cations are strongly attracted to the negative charges of MXene surfaces. The resulting site-to-site diffusion barriers within the slit (Figure 3g) substantially hinder both lateral migration along the pore walls and subsequent desorption into bulk water. This surface-binding dominated transport regime accounts for suppressed cation mobility and permeability under extreme confinement. As the channel opens to 2.7 Å, the ion-dependent barriers become more differentiated and give rise to pronounced cation selectivity. Based on the Smoluchowski-type bottleneck estimation, Mg^2+^ shows a much higher exit barrier than the other cations: 178.18 kJ mol^–1^ for Mg^2+^, compared with 112.93 kJ mol^–1^ for Ca^2+^ and 108.87 kJ mol^–1^ for Li^+^. This large exit barrier explains the strong suppression of Mg^2+^ permeation. By contrast, Ca^2+^ has an exit barrier close to that of Li^+^, indicating that it is not kinetically trapped in the same way as Mg^2+^. Together with the stronger confined-water absorbance observed for Ca^2+^, this supports a water-mediated transport pathway for Ca^2+^ in the 2.7 Å channel. As the interlayer spacing further increases, the diffusion and exit barriers are progressively reduced for all cations, and the energetic separation between ions becomes less pronounced, indicating weakened ion-specific energetic discrimination and diminished selectivity.

### Permselective Ion Transport

The ion selectivity between cations and anions was investigated using the drift–diffusion method. A schematic of the setup is shown in Figure 4a. A variable potential difference was applied between a highly concentrated salt solution and another solution with a 10-fold lower concentration (see the Experimental Section for details). For all cations, the zero-current condition occurs at negative potentials (Figure 4b), and the Li–Ti_3_C_2_T*_x_* channel exhibits lower cation mobility than anion mobility. This observation at ultralow interlayer spacing differs from previous observations for negatively charged graphene channels^39^ or for MXene with non-fixed interlayer spacing.^56,57^ The mobility ratio between cations and anions is shown in Figure 4c, as K^+^ and Cl^–^ have similar hydrated ion sizes (Supporting Table S1), and the much lower mobility of K^+^ indicates a surface charge effect on ion dehydration and mobility. Although Li^+^ naturally has lower mobility than K^+^ and Na^+^ due to its full hydration shell, the cation/anion mobility ratio for Li^+^ is higher than that for Na^+^ and slightly higher than that for K^+^, owing to water restructuring and dielectric screening effects. Additional results for different MXene interlayer spacing, surface termination, and different kinds of anions are shown in Supporting Figures S19–S20.

**Figure 4:**
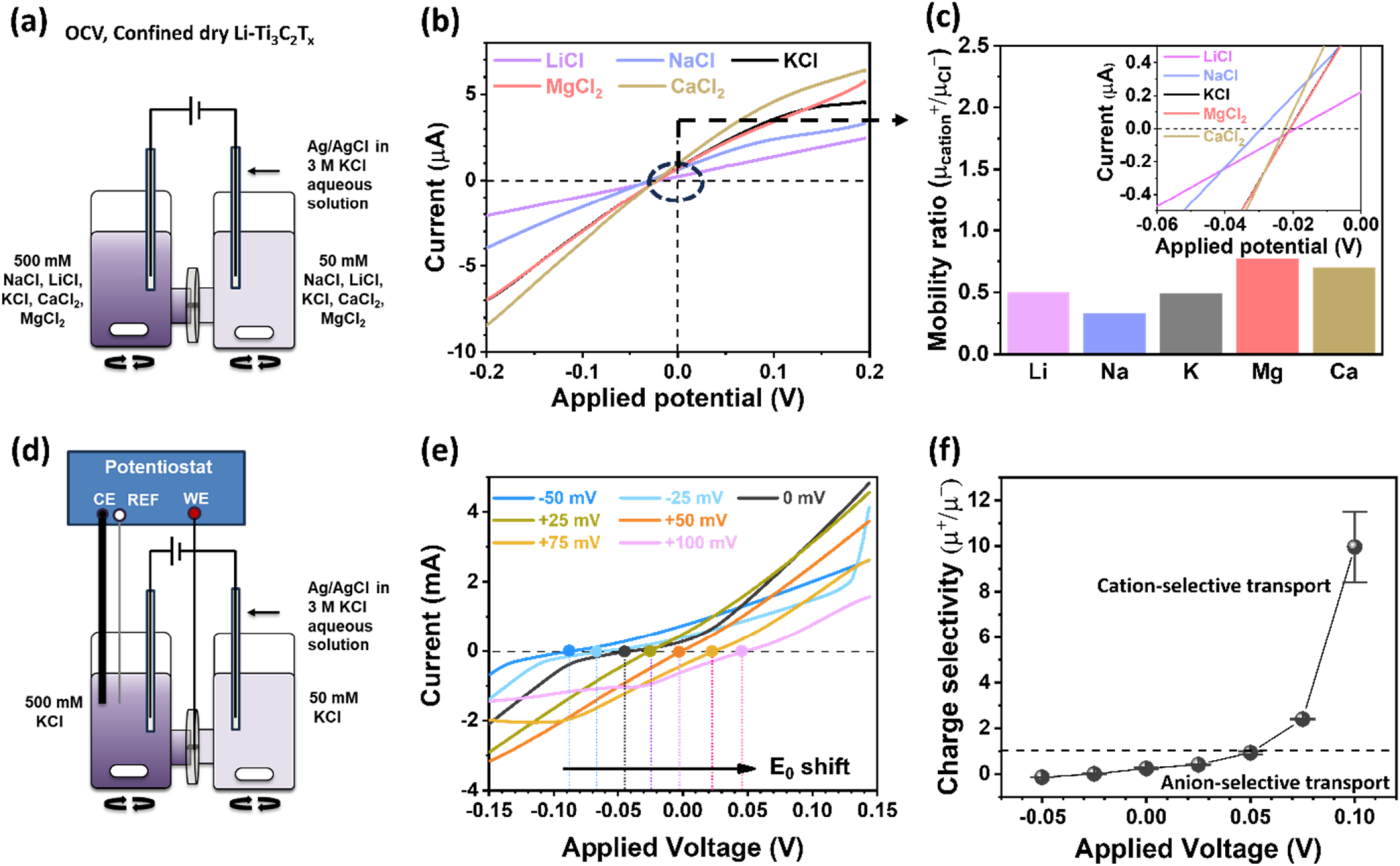
Drift-diffusion characterization (a–c) tested in different cation-containing solutions (LiCl, NaCl, KCl, MgCl_2_, CaCl_2_): (a, d) Schematic illustrations of the setup; (b) *I–V* curves (current vs applied voltage); and (c) calculated mobility ratio between cations and anions. (e, f) Electrochemical potential-controlled ion permselectivity: (e) *I–V* curves (current vs applied voltage) recorded under different potentials vs Ag/AgCl applied to the Li–Ti_3_C_2_T_*x*_ channel; (f) calculated cation/anion mobility ratio under different potentials applied to the Li–Ti_3_C_2_T_*x*_ channel.

Although our data reveal that anions exhibit higher mobility than cations within MXene nanoconfinement (Figure 4a–c), ion selectivity can arise from distinct physical mechanisms depending on the application. Selective ion transport is primarily governed by differences in mobility, whereas energy storage performance is largely dictated by selective ion uptake and adsorption at the electrode surface. In MXene systems, the strong interaction between the negatively charged surface and cations promotes preferential cation adsorption, making ionic mobility strongly dependent on the surface charge and interfacial electrostatics. To directly probe the tunability of ion transport, we varied the electrode potential applied to the MXene channel (Figure 4d–f; the setup connection is described in the Experimental Section). At 0 mV vs Ag/AgCl, the channel preferentially transports anions due to the attractive interaction between cations and the negatively charged surface. As the applied potential becomes increasingly negative, the zero-current potential *E*_0_ shifts to more negative values. As positive potentials (vs reference) are applied until above 50 mV, the zero-current potential becomes positive. The electrochemical potential modulates the electron density and surface charge within the MXene nanoconfinement, thereby switching surface–ion interactions and fundamentally altering ion permselectivity. The shift of *E*_0_ is approximately proportional to the applied potential with respect to the reference electrode. After calculating the mobility ratio between cations and anions (see the Experimental Section), we can observe a gradual change in ion permselectivity, revealing two distinct transport regimes: as a potential below 50 mV is applied to the MXene channel electrode, the Li–Ti_3_C_2_T*_x_* electrode shows selective anion transport due to higher affinity with cations; when the MXene electrode potential is above 50 mV, the electrode preferentially transports cations. Our results show high tunability in both ion selectivity and ion permselectivity in constrained MXene channels. After the drift-diffusion test with applied potential on the MXene channel, the XPS data of the postcycled film are shown in Supporting Figure S7­(e,f). There is no pronounced oxidation in the Li–Ti_3_C_2_T_*x*_ channel.

## Conclusions

We established subnanometer water confinement as a key regulator of selective ion transport in Ti_3_C_2_T*_x_* MXene channels with precisely controlled interlayer spacing between 0.9 and 5.0 Å, tunable surface terminations, and electrode potential. Li^+^ permeates nearly two orders of magnitude faster than in previous 2D membranes (∼90 mol·m^–2^ h^–1^). A hydrophobic chlorine-terminated MXene surface suppresses water transport, and the transport of most cations except for Li^+^ drops below the detection limit, thereby enhancing Li^+^ selectivity.

MD simulations reveal that the dominant energy barriers arise from diffusion within the nanoconfinement and ion exit, while the reorganization of confined water dynamically tunes ion–surface interactions, thereby explaining observed selectivity trends.

Tuning surface charge by applying potential to the MXene channel reversibly switches transport from anion- to cation-selective, achieving a K^+^/Cl^–^ mobility ratio of 12 at only 100 mV vs Ag/AgCl.

These findings establish clear design rules for subnanometer artificial ion channels and ion-selective membranes: confinement size and surface chemistry dictate hydration state and water orientation, while electrochemical potential governs ion–surface coupling. This integrated strategy provides a framework for designing high-performance membranes and ionotronic devices for selective ion separation, lithium extraction, water desalination, and electrically gated nanofluidic systems.

## Supplementary Material



## Data Availability

All data are available in the main text or the Supporting Information.

## Notes

Experimental and simulation details, methods, materials, and data, including photographs of the experimental setup (PDF)
