# Supplementary material for: Water-Mediated Ion Selectivity in 2D MXene Channels
Source: J Am Chem Soc. 2026 Jul 1;148(29):31253–62. doi: 10.1021/jacs.6c07978 (PMC13426300; doi:10.1021/jacs.6c07978)
Supplement: Supplementary file 1 [file ja6c07978_si_001.pdf]

Water-mediated ion selectivity in 2D MXene channels

Yuan Zhang[1†], Ming Chen[2†], Teng Zhang[1], Tetiana Parker[1], Danzhen Zhang[1], Ruocun Wang[1], Hyunho Kim[1], Paweł Piotr Michałowski[3], Alexei Kornyshev[2], Yury Gogotsi[1]*

[1]A.J. Drexel Nanomaterials Institute and Department of Materials Science and Engineering, Drexel University, Philadelphia, PA 19104, United States

[2] Department of Chemistry, Faculty of Natural Science, Imperial College London, Molecular Sciences Research Hub, White City Campus, Wood Lane, London,W12 0BZ, UK

[3] Łukasiewicz Research Network—Institute of Microelectronics and Photonics, Warsaw, Poland

Experimental

MAX phase synthesis

TiC (Alfa Aesar, 99.5%, 2 µm powder), Ti (Alfa Aesar, 99.5%, 325 mesh), and Al (Alfa Aesar, 99.5%, 325 mesh) powders (2:1.25:2.2 atomic ratio) were mixed and ball-milled with zirconia balls for 18 h at 70 rpm [1]. Subsequently, the powder mixture was transferred to alumina crucibles and sintered at 1380 °C for 2 h with a heating rate of 3 °C/min under 100 SCCM Ar flow. The sintered MAX phase was milled into powder and washed with 9 M HCl until gas bubbles ceased. The MAX phase was then neutralized with deionized water and dried at 80 °C overnight.

MXene synthesis

$Ti_3C_2T_x$ was synthesized by selectively etching of Ti_3AlC_2 MAX phase powders (325 mesh) using a mixture of HF (48.5–51%, Acros Organics) and HCl (36.5–38%, Fisher Chemical) acids. 2 ml of HF, 12 ml of HCl and 6 ml of de-ionized (DI) water were mixed first following by 1 g of MAX phase powder was added to the solution and stirred for 24 h at 35 °C. The reaction product was washed with DI water by centrifugation at 2550 g for 2 min until pH > 6. The resulting sediment was dispersed in a 0.5 M LiCl solution, shaken for 15 min, and then centrifuged at 2550 g for 10 min several times until the sediment delaminated and swelled. The swollen sediment was dispersed in DI water and then centrifuged at 2550 g for 10 min. The dark supernatant was collected for spray-coating to fabricate electrodes.

$Ti_3C_2Cl_2$ synthesis

Cl-terminated MXene was synthesized via a modified Lewis acid molten salt (LAMS) method, previously reported [2,3]. $ZnCl_2$ was used as the Lewis acid for $Ti_3C_2Cl_2$ synthesis, and powder handling and mixing were performed in an Ar-filled glovebox. Ti_3AlC_2 MAX phase (10 g) and $ZnCl_2$ (80 g) were thoroughly mixed, transferred to an alumina crucible, and heated to 640 °C at 5 °C min^{-1} under Ar flow for 4 h. After cooling, the product was washed in 12 M HCl to remove residual $ZnCl_2$ and Zn byproducts, followed by multiple DI water washes and centrifugation to remove small flakes. The multilayer MXene was filtered through a 5 µm polycarbonate membrane, briefly air-dried, and further dried under vacuum at 45 °C for 48 h. The resulting MXene powders were stored in an inert atmosphere for subsequent use.

$Ti_3C_2Cl_2$ delamination

Delamination was performed in an argon-filled glovebox to maintain a moisture-free environment [4]. First, an intercalation solution was prepared by dissolving 1.2 g of lithium chloride (LiCl) in 10 mL of anhydrous dimethyl sulfoxide (DMSO, Sigma Aldrich, >99.9%) in a 20 mL glass vial. The solution was stirred with a PTFE-coated magnetic stir bar for 10 minutes until the salt fully dissolved. Subsequently, 3 g of multilayer LAMS $Ti_3C_2Cl_2$ powder was added to the solution and stirred at 1,000 rpm for 24 hours to facilitate Li-ion intercalation.

After intercalation, the mixture was transferred to a 15 mL centrifuge tube and centrifuged at 1,500 rcf for 5 minutes to remove the supernatant. To remove excess LiCl and residual DMSO, the

sediment was washed twice with 10 mL of anhydrous tetrahydrofuran (THF, Sigma Aldrich, >99.9%). For each wash, the mixture was centrifuged at 1,500 rcf for 5 minutes, and the supernatant was decanted.

To induce swelling, the washed sediment was redispersed in anhydrous N-methylformamide (NMF, Sigma Aldrich, >99.9%). The sediment was divided into two vials, each containing 15 mL of NMF. Intense manual shaking was used to redisperse the sediment, which exhibited a slurry-like morphology due to volume expansion. The dispersion was centrifuged, and the dark brown supernatant containing small multilayers was discarded. This NMF washing process was repeated until the sediment swelled to approximately five times its original volume.

Final delamination was achieved by redispersing the NMF-swelled sediment in anhydrous DMSO. The dispersion was centrifuged at 250 rcf for 5 minutes. The resulting dark supernatant, containing large flakes of delaminated $Ti_3C_2Cl_2$, was collected. This DMSO extraction was repeated multiple times until the supernatant became transparent. To maximize yield, the residual sediment was subjected to bath sonication (Branson 2501 Ultrasonic Cleaner, 40 kHz) in DMSO for 20 minutes in a sealed tube under argon, followed by collection under ambient conditions. Finally, the collected colloidal solution was concentrated by centrifugation at 11,200 rcf for 10 minutes. The resulting sediment was redispersed in fresh anhydrous DMSO and collected for future use.

Channel preparation

Microscopic 8 × 8× 1 mm glass slides (Fisher Scientific) were cleaned by sequential bath sonication (2510 Ultrasonic Cleaner, Branson) in detergent solution (Hellmanex III, Fisher Scientific) to remove residue from the glass surface. Subsequently, they were immersed in deionized water and ethanol for 5 min, sonicated, and dried with compressed air. The cleaned slides were plasma treated (Tergeo Plus, Pie Scientific) at 100 W with Ar/O_2 at 3/5 SCCM for 5 min to make the surface hydrophilic. The pre-treated slides were spray-coated with $Ti_3C_2T_x$ suspension and air-dried with a hair dryer (1875, Conair). The $Ti_3C_2T_x$ suspension used for spray-coating has a concentration of approximately 5 mg ml^{-1}, resulting in spray-coated films with a thickness of ~50-100 nm. The thickness of each spray-coated film was measured using laser scanning confocal microscopy (Keyence, VK-X1000).

After overnight drying of the spray-coated film in a desiccator, Li-$Ti_3C_2T_x$ is obtained. To exchange or add intercalants within the $Ti_3C_2T_x$ confinement, 5 M HCl, deionized water, and 2 mM TMAOH solutions were dropped onto the Li-$Ti_3C_2T_x$ to wet the film for 5 min. The liquid residue was carefully absorbed by Kimwipes tissue. The obtained wet films were then placed in a vacuum oven at 80 °C for 12 h to dry. The dried films were stored in a desiccator. $Ti_3C_2Cl_2$ MXene film was obtained by drop casting on the glass substrate and dried in a vacuum oven at 80 °C for three days.

For channel fabrication, one drop of epoxy resin and hardener (from ALLIED) mixed at a mass ratio of 100:12 was cast on top of a film covered by another MXene film of the same kind, layer-by-layer. Three layers of film were stacked and constrained by epoxy. The top layer of film was constrained and covered by a glass slide of the same size. The epoxy mixture covered each MXene channel and filled all uneven spaces and microvoids, but left the nanoconfinement area open. The constrained channels were ready once the epoxy solidified overnight. The fabricated channels were fixed into an acrylic plate with epoxy, as shown in Fig. 1a, and then dipped into deionized water overnight before conducting ion-selective permeation and drift-diffusion tests.

Ion-selective permeation test

The ion permeation experiment was conducted using 100 mM of LiCl, NaCl, KCl, $MgCl_2$, or $CaCl_2$ as the feed solution, and deionized water as the permeate solution. The two solutions were separated by an acrylic plate with horizontally aligned MXene channels, and stirred for 48 h to allow ions diffuse from the feed solution to the permeate solution. Ion chromatography and elemental characterization were performed at the permeate side using Metrohm Eco IC and Agilent 5900 ICP-OES for double-checking.

The ion permeation rate was obtained by calculating the mass transport number divided by the $Ti_3C_2T_x$ film cross-section and time from the following equation:

$$Ion\ permeation\ rate = \frac{C_{initial} - C}{t_n - t_{n-1}} * \frac{V_{Permeate}}{A_{Channel}} \qquad (1)$$

where C_{initial} is the initial concentration. In our work, we applied deionized water as initial permeate, so C_{initial} for all cations is zero. C is the ion concentration measured by ion chromatography elemental characterization, (t_{n}–$t_{\text{n–1}}$) is the sampling period, V_{permeate} is the volume of the permeate solution, and A_{Channel} is the cross-section of the constrained MXene film ion channel. Ion selectivity is defined here as the ratio of permeation rates between two ionic species.

Secondary-ion mass spectrometry (SIMS) characterization

SIMS measurements were performed without dismantling the sample. Instead, narrow access craters were locally milled through the glass encapsulation using a high-energy cesium ion beam (16 keV, 700 nA). This approach follows the methodology proposed by Michałowski [5], which allows precise targeting of buried structures without compromising sample integrity. Each crater had a size of 500 × 200 μm to prevent shadowing effects during deep profiling.

Following this initial milling, SIMS analysis was conducted with a lower-energy beam (5 keV, 5 nA). Measurements were taken at three depths: the interface between encapsulation (glass or PET) and the MXene membrane, the central region of the membrane, and the interface between the membrane and the glass substrate. Additionally, six lateral positions along the membrane length were probed: inlet, outlet, and 20%, 40%, 60%, and 80% of the total channel length.

Cesium was used as the primary ion species with positive detector polarity. Thus, all elements were analyzed as molecular secondary ions of the type CsX^+ and subsequently normalized to the Cs^+ signal to improve quantification.

Fourier Transform Infrared (FTIR) characterization

FTIR spectroscopy was used to analyze the vibrational spectra of MXene channels in different salt solutions. Spectra were processed using concave rubberband correction and 25-point average smoothing. The spectral region between 2700-2000 cm^{-1} was excluded due to interference from the ATR crystal signal [6]. For FTIR characterization, one layer of constrained MXene film was prepared for all sample types as described in the Channel Preparation section. The fabricated channel was soaked in a mixed water solution of 0.1 M LiCl, NaCl, KCl, $MgCl_2$, and $CaCl_2$ for 48 h. Before each FTIR characterization, the channels were taken out of the solutions, and excess water outside the channels was removed with Kimwipes. The cover glass and epoxy were peeled off and the MXene films were exposed immediately before characterization. The MXene films on glass substrates were then placed face down and attached to the ATR crystal to let the IR beam directly shine into the MXene confinements.

Molecular dynamics simulation

MD simulations were utilized with the GROMACS package [7]. Specifically, we employed MD simulation of aqueous electrolytes confined between MXene, as shown in (Supplementary Fig. S9) the SPC/E model was adopted for water [8]. The force field parameters for ions were taken from OPLS [9].The interelectrode distance was set to 10 nm, ensuring a bulk-like electrolyte region in the channel center.

All simulations were performed in the NVT ensemble. Temperature was controlled through the v-rescale thermostat [10] at 300 K with a coupling constant of 1.0 ps. A cutoff distance of 1.2 nm was employed for the van-der-Waals term via direct summation. Long-range electrostatic interactions were calculated using the Particle Mesh Ewald (PME) method [11]. An FFT grid spacing of 0.1 nm in conjunction with cubic interpolation was employed for computing the reciprocal space electrostatic interaction. Specifically, to accurately account for the electrode polarization effects in the presence of electrolytes, the constant potential method (CPM) was implemented to allow for the fluctuations of the charges on electrode atoms [12,13]. Each simulation began with a heating phase at 400 K for 3 ns and then annealed to 300 K over 2 ns, followed by another 5 ns to reach equilibrium. Thereafter, a 15 ns production was performed for analysis. Each case was repeated three times with varying initial configurations to certify the accuracy of the simulation.

Ordering Parameter. The orientational ordering of confined water molecules was quantified by analyzing the probability distribution of the angle θ between the water dipole moment and the

vector pointing from the oxygen atom of the water molecule to the neighboring ion (e.g., Li^+). For each water molecule i, the orientational angle was defined through:

$$\cos\theta_i = \boldsymbol{\mu_i} \cdot \boldsymbol{r}_i \quad (2)$$

where $\boldsymbol{\mu_i}$ is the unit vector along the dipole moment of the water molecule, and $\boldsymbol{r}_i$ is the unit vector pointing from the oxygen atom to the ion. The first-order orientational order parameter was then calculated as [14]

$$P_1 = \int p(\theta)\cos\theta\, d\theta \quad (3)$$

which corresponds to the ensemble-averaged projection of the water dipole onto the ion–water radial direction. A value of P_1 approaching 1 indicates a highly ordered solvation structure in which water dipoles are strongly aligned along the ion–water radial direction, corresponding to a stable and mobile hydration shell.

PMF calculation. Potential of mean force (PMF) calculations were carried out to resolve free-energy barriers associated with ion intercalation, in-plane diffusion, and pore exit. The PMF was obtained using the umbrella sampling method [15] ,in which a harmonic biasing potential was applied to restrain the ion at predefined positions along the transport coordinate. For each sampling window, the ion was allowed to move freely in the xy direction, ensuring proper sampling of lateral configurations. The resulting free-energy profiles were reconstructed using the weighted histogram analysis method (WHAM) [16].

Drift diffusion characterization

The setup is shown in Fig. 4(a,d). The two reservoirs were filled with salt solutions containing different cations, with a concentration gradient of ΔC = 10 (500 mM and 50 mM in opposite reservoirs). Two Ag/AgCl electrodes (both filled with 3 M KCl, to avoid Nernst potential contribution) were applied as the two electrodes were immersed in two reservoirs . The electrode on the lower concentration side was connected to the working electrode, and the electrode on the high concentration side was connected to the counter electrode. A linear potential scan at a scan rate of 1 mV/s was applied to the two electrodes (from +0.2 V to -0.2 V).

For drift-diffusion characterization under controlled potential, KCl solutions of 500 mM (feed) and 50 mM (permeate) were used. Two potentiostat channels were employed using a BioLogic VMP-300. A variable potential difference was applied between two Ag/AgCl electrodes within the KCl reservoirs, connected to the first channel. The constrained Li-$Ti_3C_2T_x$ channel was extended via silver wires beyond the mounted setup and served as the working electrode. A separate silver wire served as the reference electrode, while a graphite rod was used as the counter electrode. These three electrodes were connected to the second channel, enabling precise potential control of the constrained Li-$Ti_3C_2T_x$ channel.

The zero-current potential was identified at the point where the I-V curve intersected the x-axis. Its non-zero value signifies a difference in mobility between cations and anions. A higher zero-

current overpotential from the reference zero point suggests a greater disparity in ion mobilities. This mobility ratio can be determined using the following equation [41]:

$$\frac{\mu^+}{\mu^-} = -\frac{Z_+ \ln(\Delta C) - Z_- F E_m / RT}{Z_- \ln(\Delta C) - Z_+ F E_m / RT} \quad (4)$$

where z^+ and z^- are the valence numbers of the charged species, ΔC is the concentration gradient between the feed and permeate solutions, in our case, ΔC=10. F is the Faraday constant. E_m is the zero current potential obtained from the *I-V* curve.

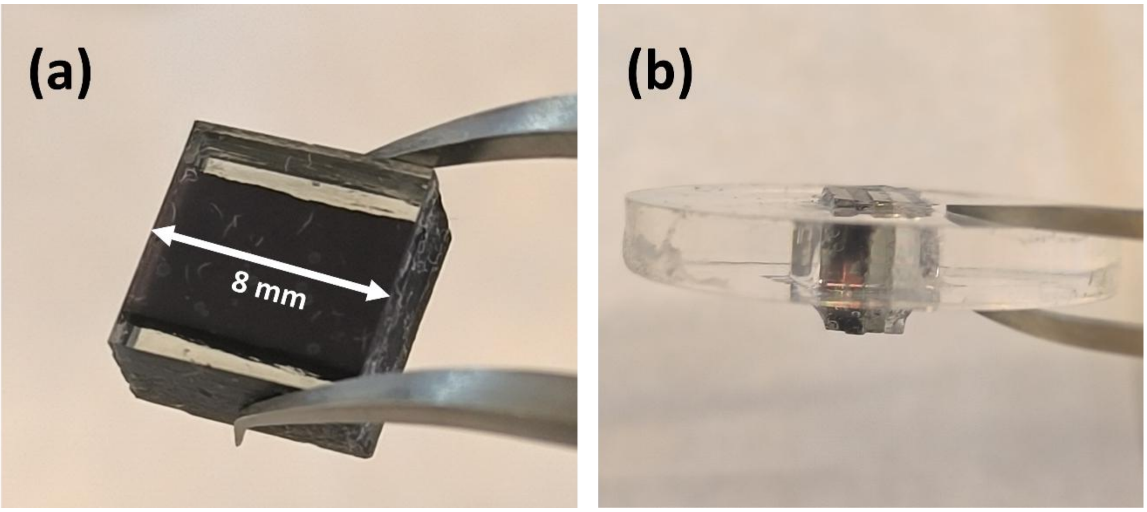

Supplementary Fig. S1. Pictures of the constrained MXene channel. (a) Stacked 3 layers of MXene channels; **(b)** MXene channel sealed in an acrylic plate using epoxy.

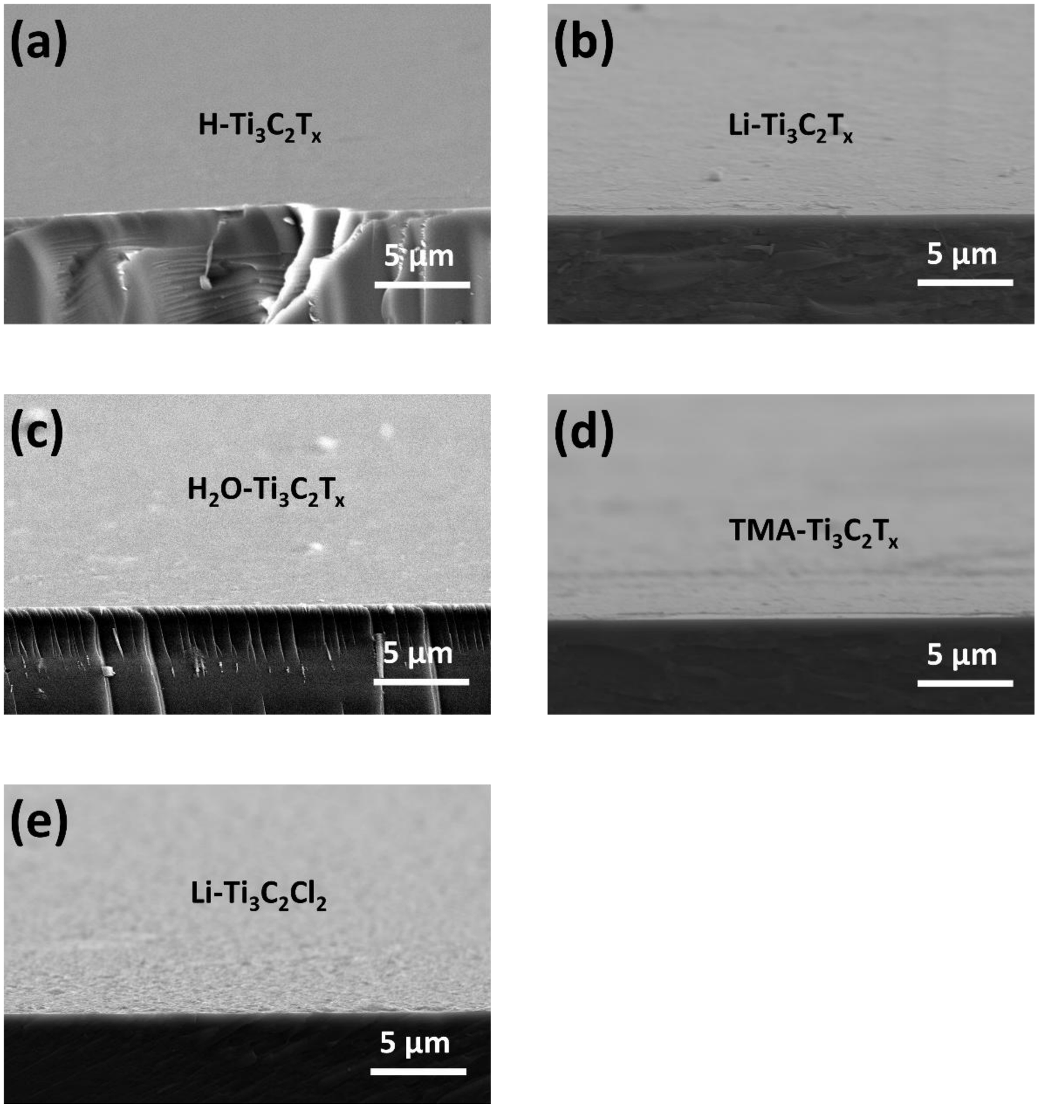

Supplementary Fig. S2. SEM images showing the surface morphologies of MXene films. (a) H-$Ti_3C_2T_x$; **(b)** Li-$Ti_3C_2T_x$; **(c)** H_2O-$Ti_3C_2T_x$; **(d)** TMA-$Ti_3C_2T_x$; **(e)** Li-$Ti_3C_2Cl_2$.

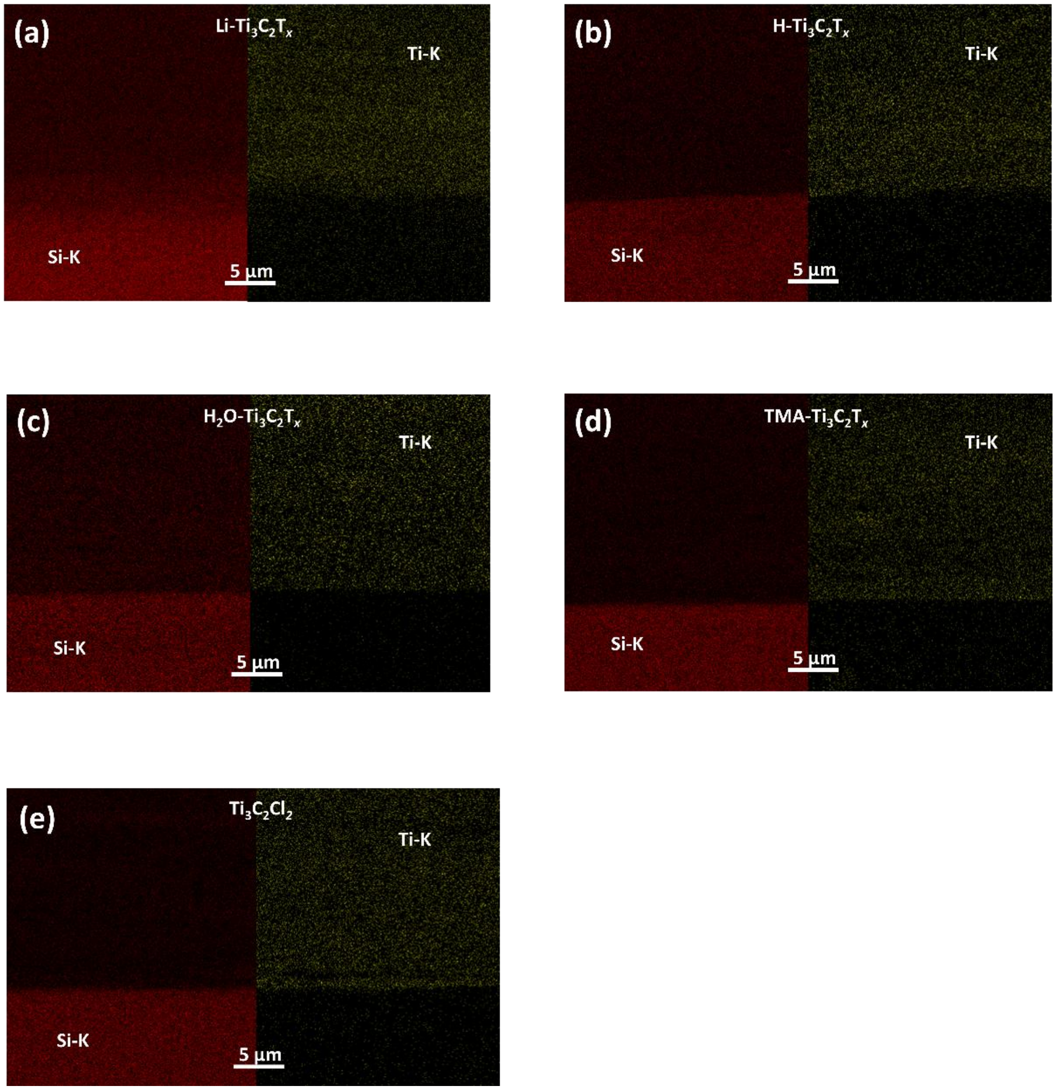

Supplementary Fig. S3. EDS elemental maps of the MXene films on glass substrates. (a) Li-$Ti_3C_2T_x$; **(b)** H-$Ti_3C_2T_x$; **(c)** H_2O-$Ti_3C_2T_x$; **(d)** TMA-$Ti_3C_2T_x$; **(e)** Li-$Ti_3C_2Cl_2$.

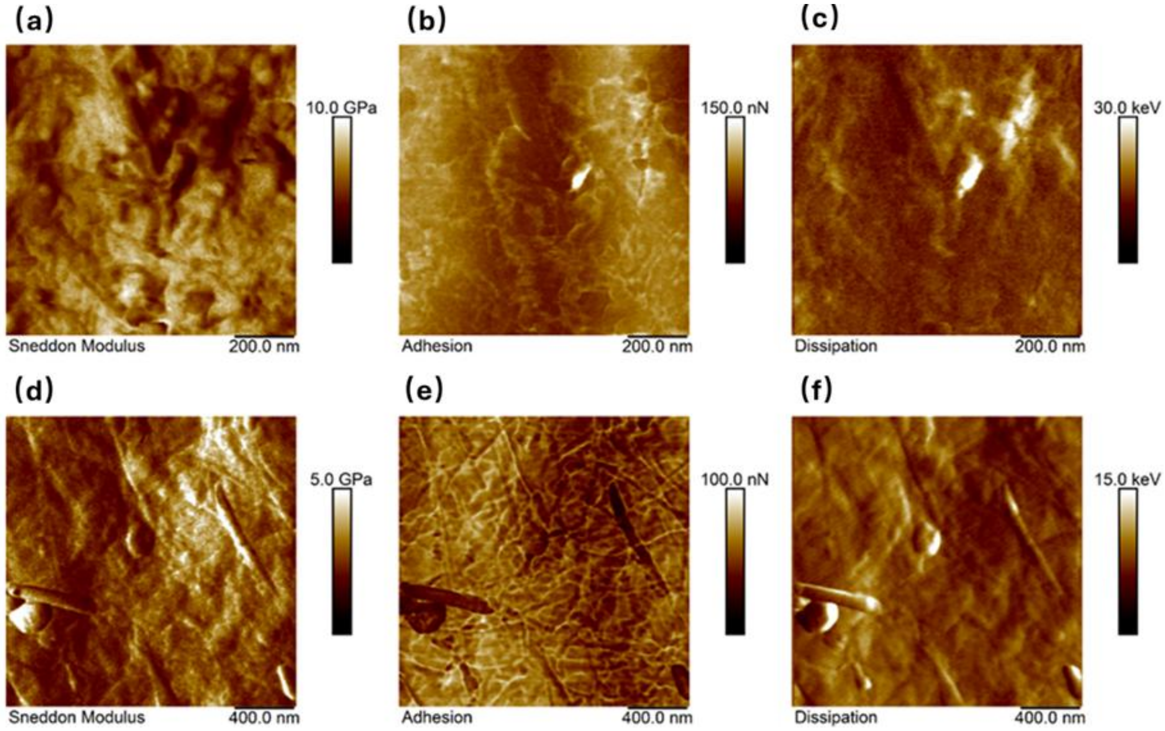

Supplementary Fig. S4. Mechanical property characterization (stiffness maps) obtained by Atomic Force Microscopy. (a-c) Li-$Ti_3C_2T_x$ and **(d-f)** Li-$Ti_3C_2Cl_2$ free-standing film.

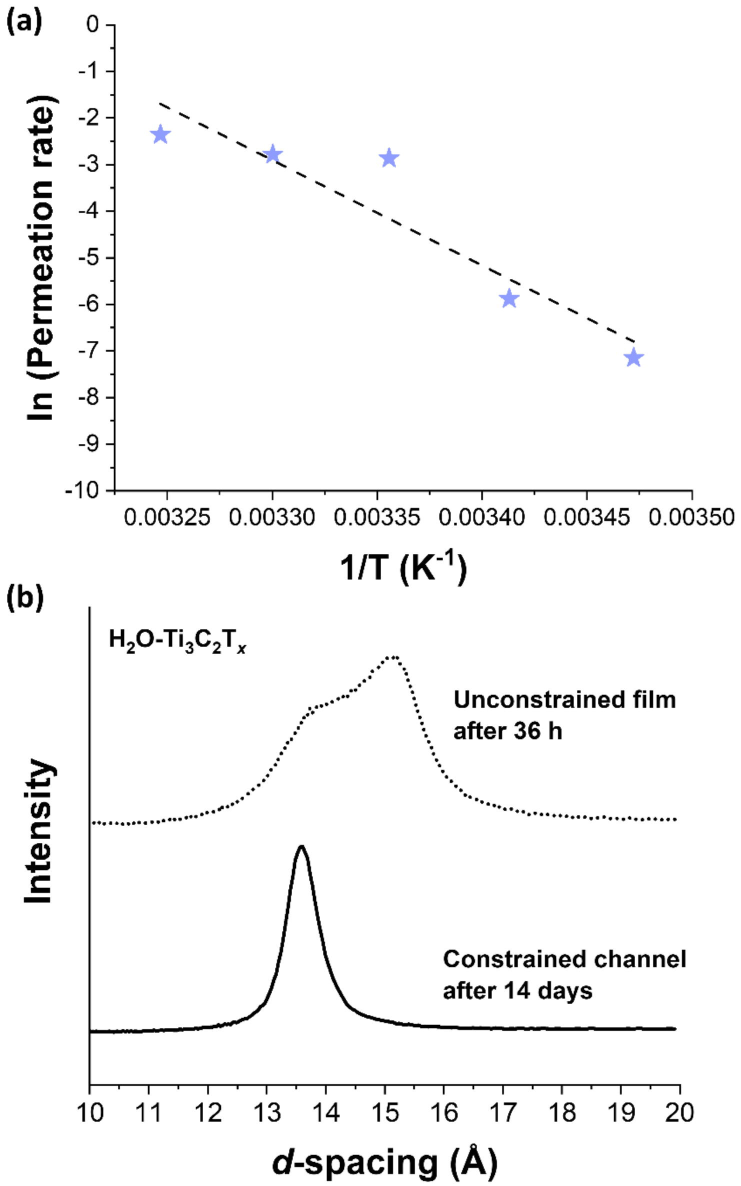

Supplementary Fig. S5. Li^+ permeation rate as a function of 1/T and structural stability of constrained H_2O-$Ti_3C_2T_x$ channel. (a) Li^+ permeation rate was measured via forward osmosis (from 1 M LiCl to Milli-Q water) under different temperatures; **(b)** Comparison of structural stability between unconstrained spray-coated H_2O-$Ti_3C_2T_x$ film and mechanically constrained channel stored in feed solution. The constrained channel design effectively suppresses structural swelling of MXene film in mixed cation-contained solution for 14 days.

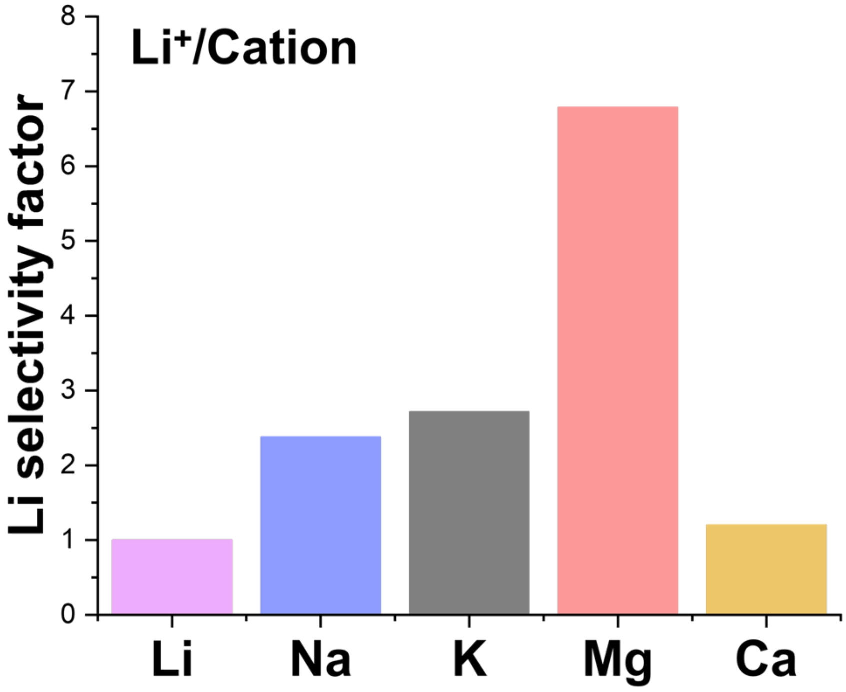

Supplementary Fig. S6. Li^+ selectivity vs all other cations during the ion permeation measurement within the Li-$Ti_3C_2T_x$ channel.

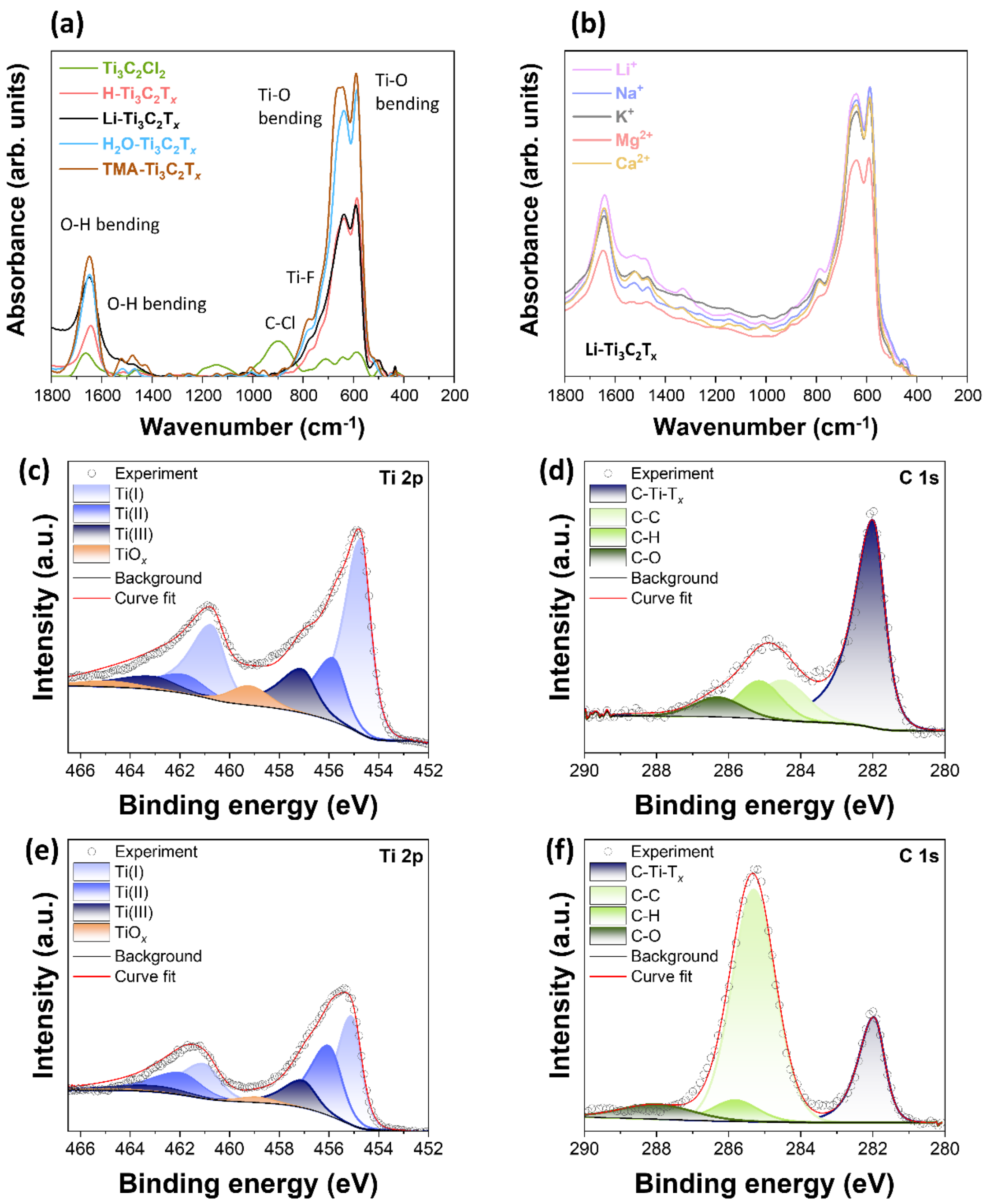

Supplementary Fig. S7. Chemical information of the constrained MXene channel. (a,b) Fourier-Transform Infrared (FTIR) spectra from the fingerprint region under different confinement environments. (**a**) With mixed cation transport (in 0.1 M LiCl, NaCl, KCl, $MgCl_2$, $CaCl_2$ aqueous solution) under different interlayer spacing pillared by intercalants; (**b**) With different single cation transport within the Li-$Ti_3C_2T_x$ channel. **(c-d)** X-ray Photoelectron Spectroscopy (XPS) of the constrained H_2O-$Ti_3C_2T_x$ channel, prepared two years ago and soaked in feed solution for 14 days. **(c)** Ti 2p, **(d)** C 1s, **(e)** O 1s. **(e-f)** X-ray Photoelectron Spectroscopy (XPS) of the constrained Li-$Ti_3C_2T_x$ channel after drift-diffusion experiments with applied potentials.

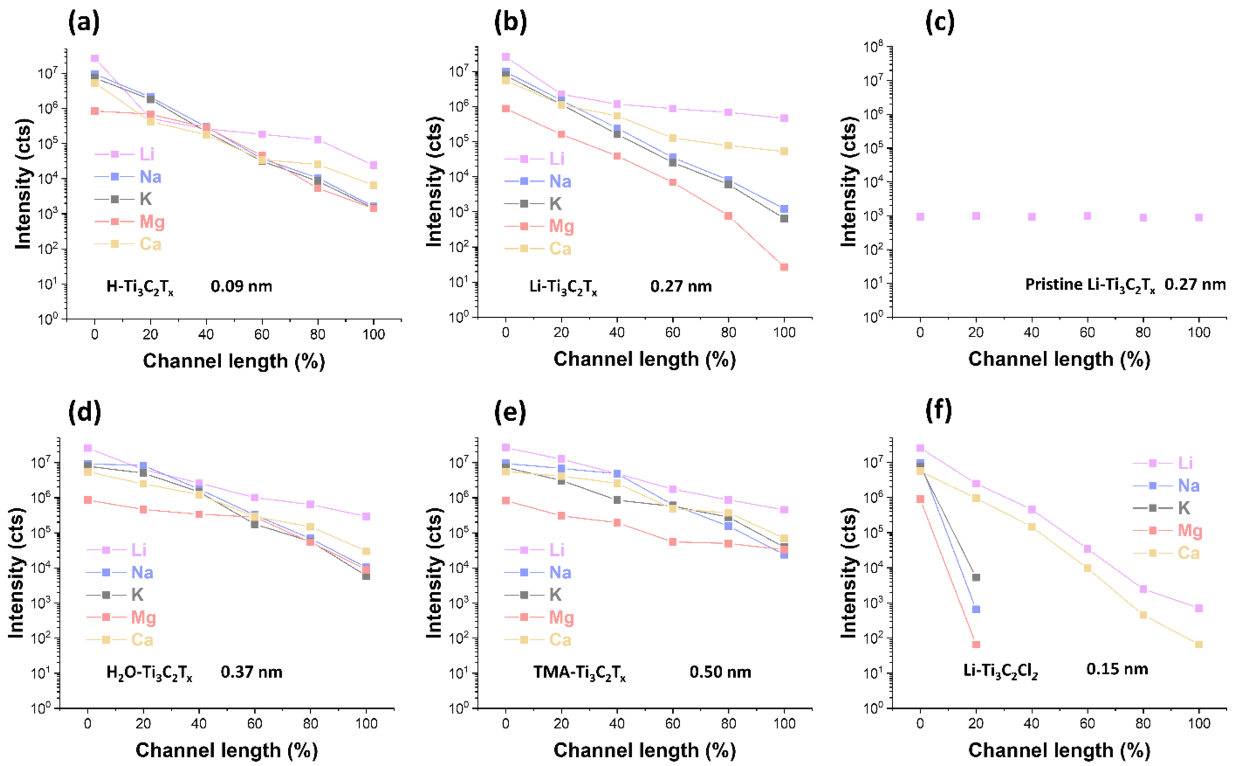

Supplementary Fig. S8. Secondary-ion mass spectrometry (SIMS) characterization of constrained MXene ion channels at different lengths. (a) H-$Ti_3C_2T_x$; **(b)** Li-$Ti_3C_2T_x$; **(c)** Pristine Li-$Ti_3C_2T_x$ without ion transportation prior to SIMs measurement; **(d)** H_2O-$Ti_3C_2T_x$; **(e)**TMA-$Ti_3C_2T_x$; **(f)** Li-$Ti_3C_2Cl_2$.

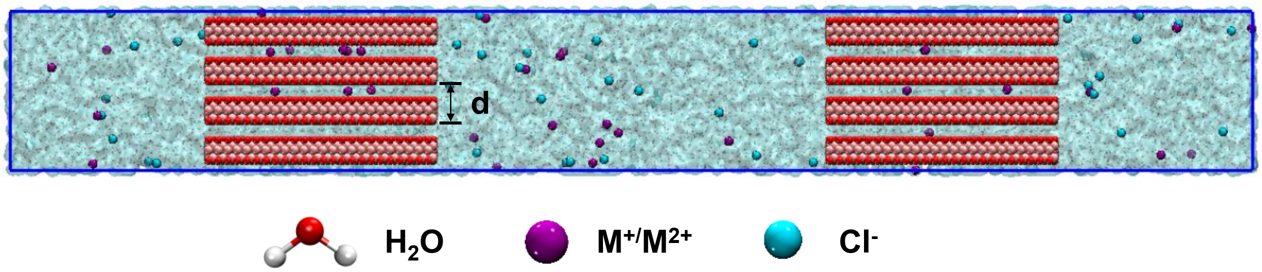

Supplementary Fig. S9. Schematics of the molecular dynamics simulation system setup.

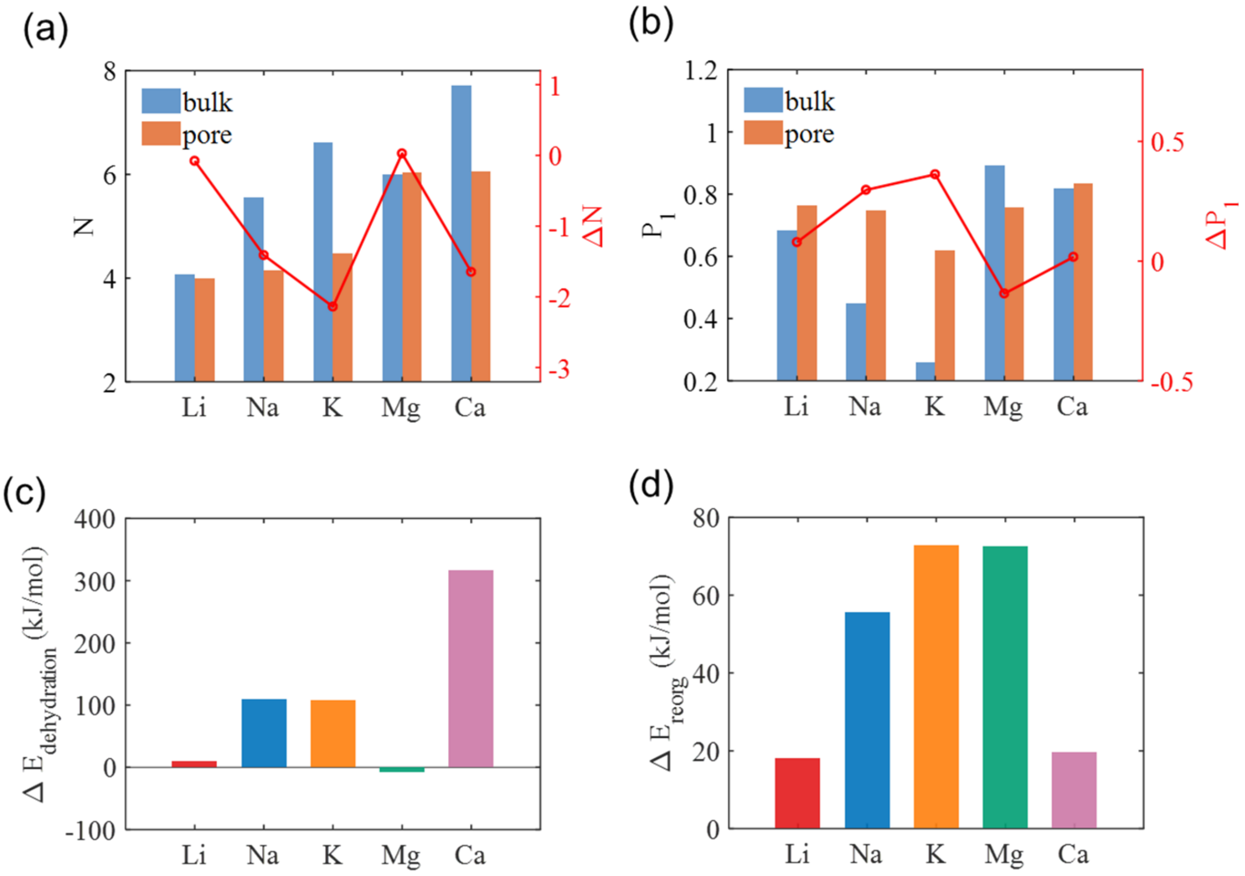

Supplementary Fig. S10. Water-mediated microscopic mechanism of selective ion transport in a 3.7 Å MXene nanochannel. (a-b) Evolution of hydration-shell structure **(a)** and first-order orientational order parameter P_1 **(b)** for different cations upon entering the MXene slit. **(c-d)** Decomposition of the energetic penalties associated with ion dehydration ($\Delta E_{\mathrm{dehydration}}$) **(c)** and confined-water reorganization ($\Delta E_{\mathrm{reorg}}$) **(d)** for different cations.

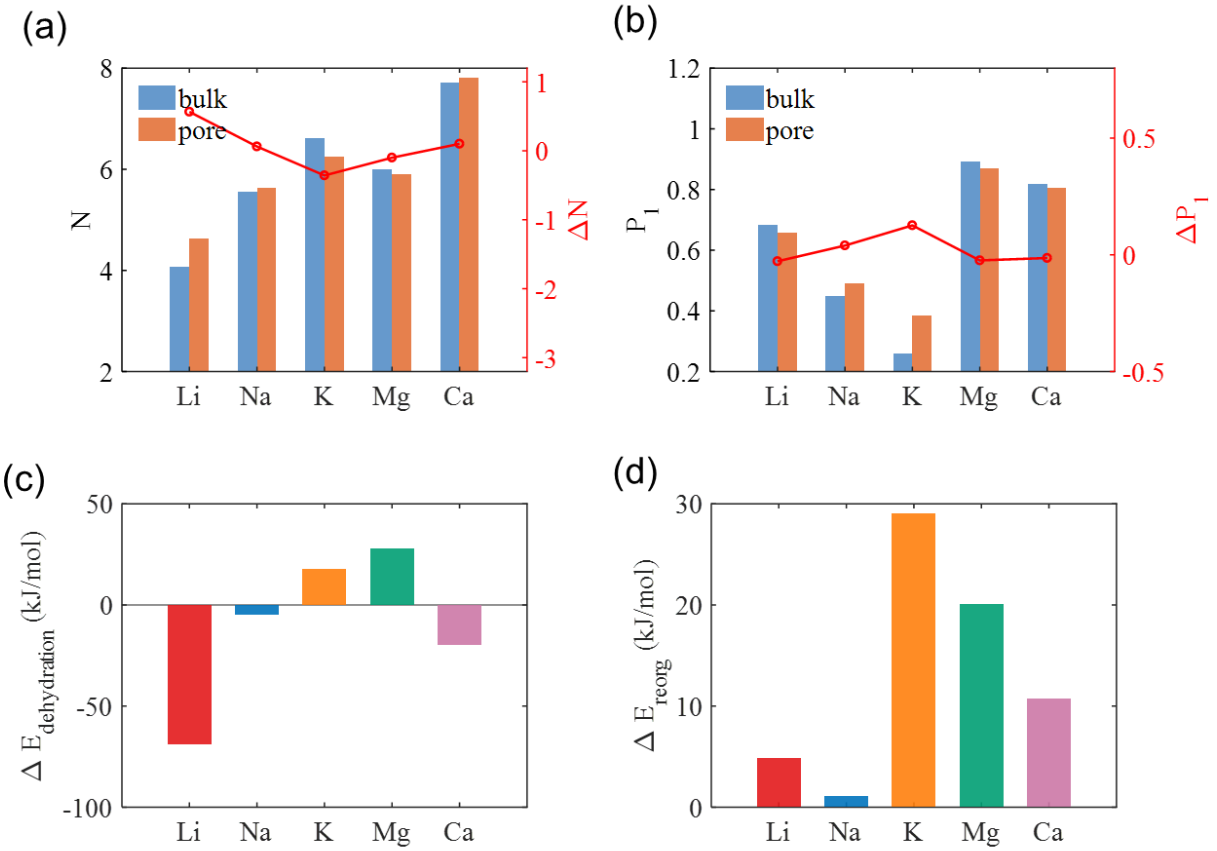

Supplementary Fig. S11. Water-mediated microscopic mechanism of selective ion transport in a 5.0 Å MXene nanochannel. (a-b) Evolution of hydration-shell structure **(a)** and first-order orientational order parameter P_1 **(b)** for different cations upon entering the MXene slit. **(c-d)** Decomposition of the energetic penalties associated with ion dehydration ($\Delta E_{\mathrm{dehydration}}$) **(c)** and confined-water reorganization ($\Delta E_{\mathrm{reorg}}$) **(d)** for different cations.

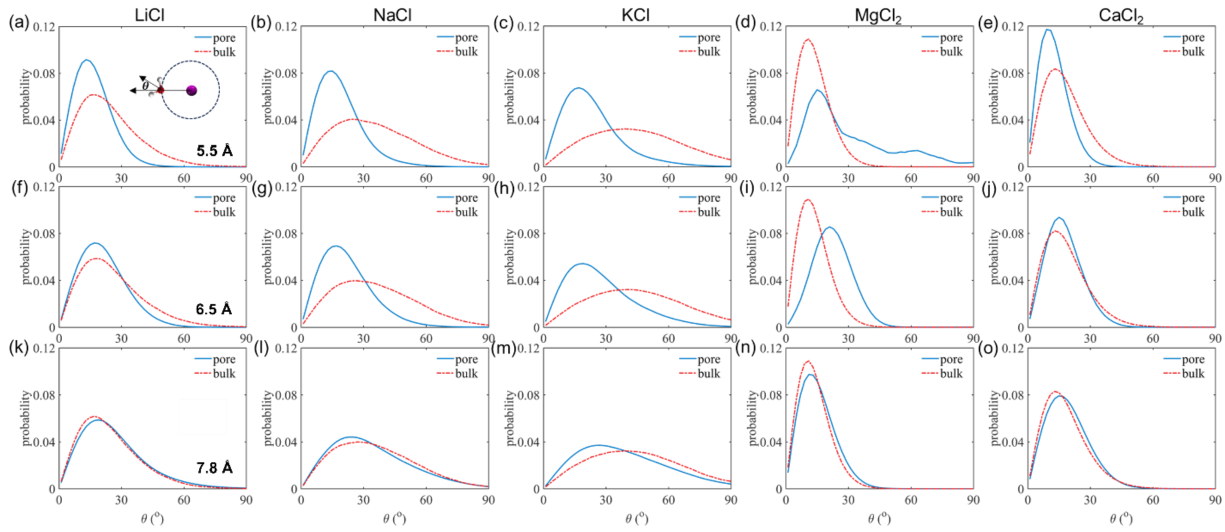

Supplementary Fig. S12. Confinement-induced changes in cation solvation structure. Orientation distributions of water molecules in the first solvation shell of cations inside MXene nanochannels, together with the corresponding bulk reference. The first solvation shell is defined by the first minimum of the cation–oxygen radial distribution function. Results are shown for MXene nanochannels with interlayer spacings of 2.7 Å (top row), 3.7 Å (middle row), and 5.0 Å (bottom row). The orientation of solvent dipoles is quantified by the angle θ between the dipole moment of water and the radial vector connecting the cation and the oxygen atom.

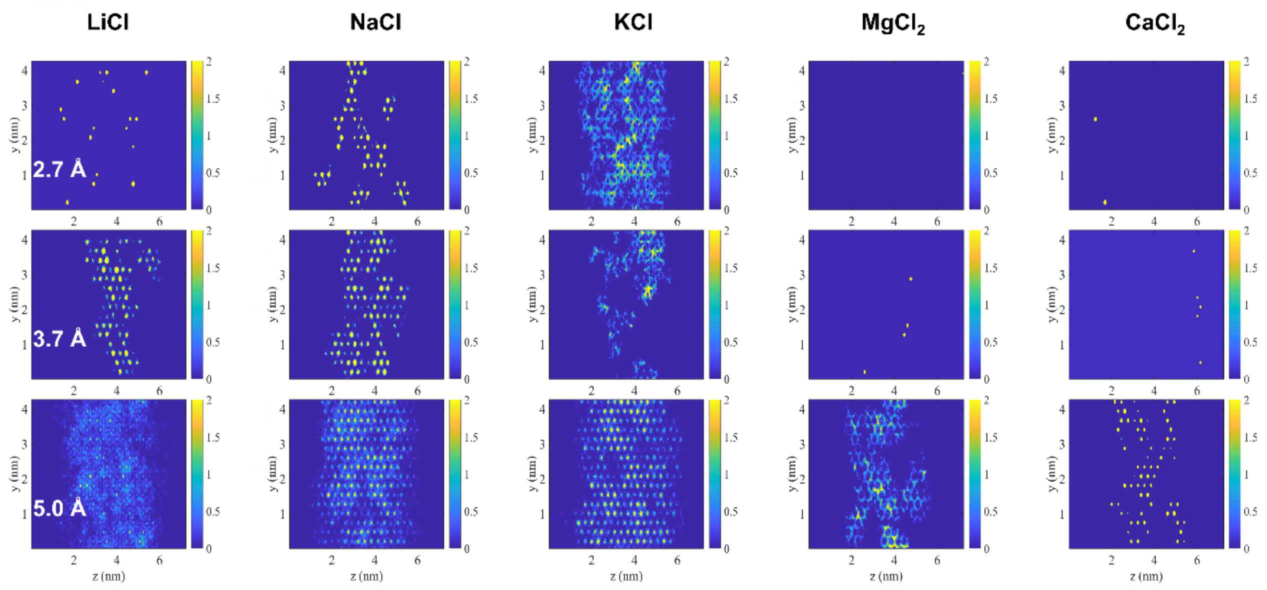

Supplementary Fig. S13. Two-dimensional in-plane number density distributions of cations inside MXene nanochannels. Results are shown for MXene nanochannels with interlayer spacings of 2.7 Å (first row), 3.7 Å (second row), and 5.0 Å (third row). The color scale represents the number density in units of nm^{-3}.

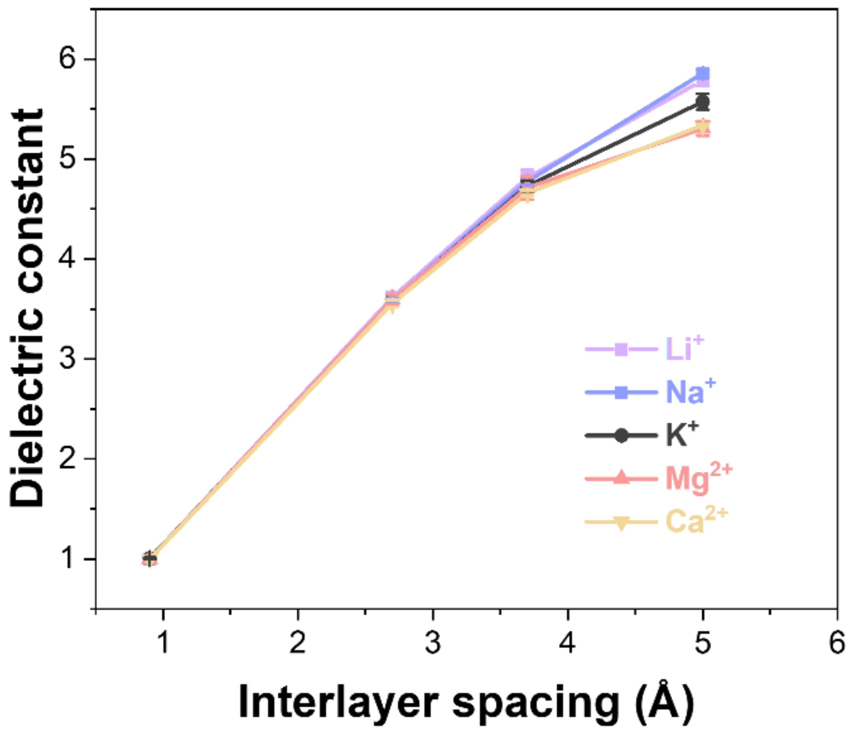

Supplementary Fig. S14. Dielectric constant of MXene channel confined water under different interlayer spacing with different cations.

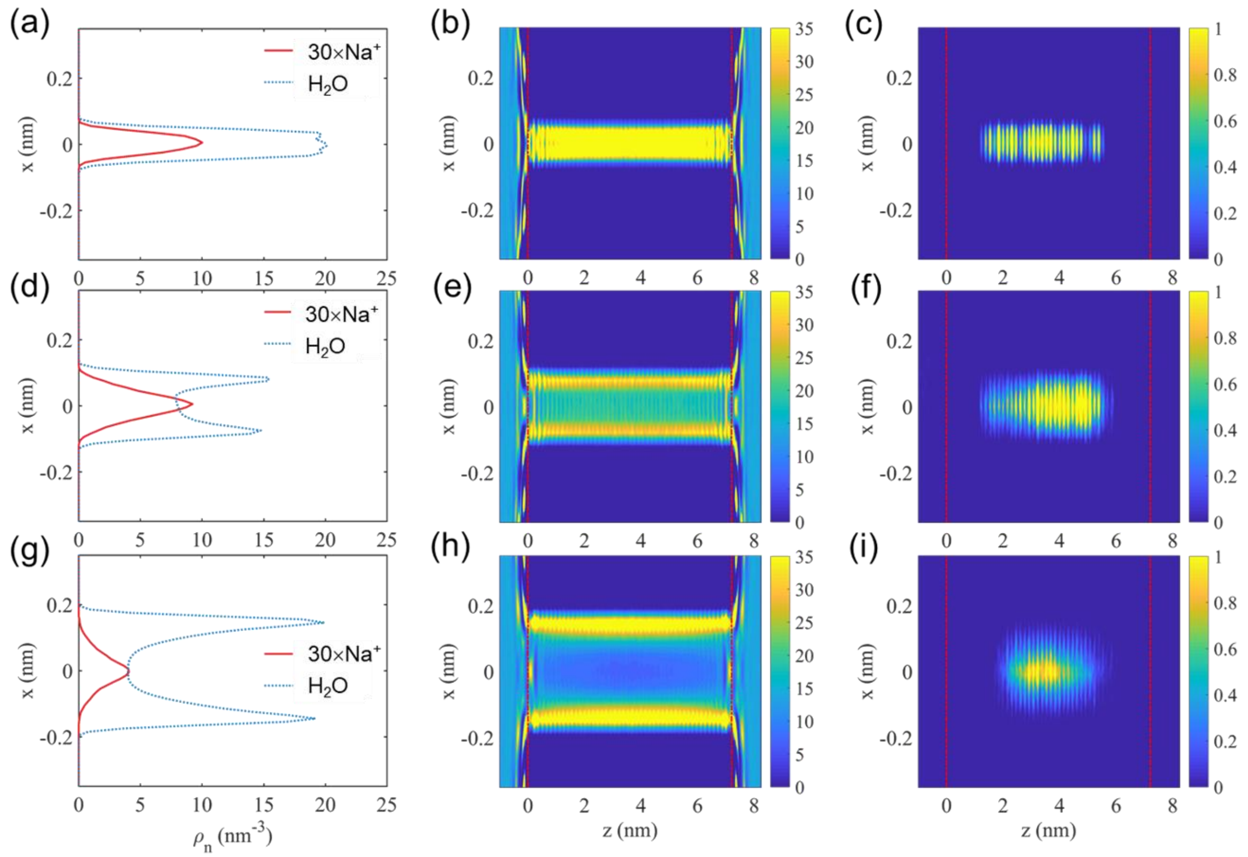

Supplementary Fig. S15. Number density distributions of cations and water molecules inside MXene nanochannels. (a,d,g) One-dimensional number density profiles of Na^+ ions and water molecules along the confinement direction normal to the MXene plane (x-direction) for nanochannels with interlayer spacings of 2.7 Å (first row, **a**), 3.7 Å (second row, **d**), and 5.0 Å (bottom row, **g**). The x-direction is defined as the direction normal to the MXene basal plane (confinement direction). (**b,e,h**) Two-dimensional number density maps of water molecules in the x–z plane for MXene nanochannels with interlayer spacings of 2.7 Å (**b**), 3.7 Å (**e**), and 5.0 Å (**h**). (**c,f,i**) Two-dimensional number density maps of Na^+ ions in the x–z plane for MXene nanochannels with interlayer spacings of 2.7 Å (**c**), 3.7 Å (**f**), and 5.0 Å (**i**).

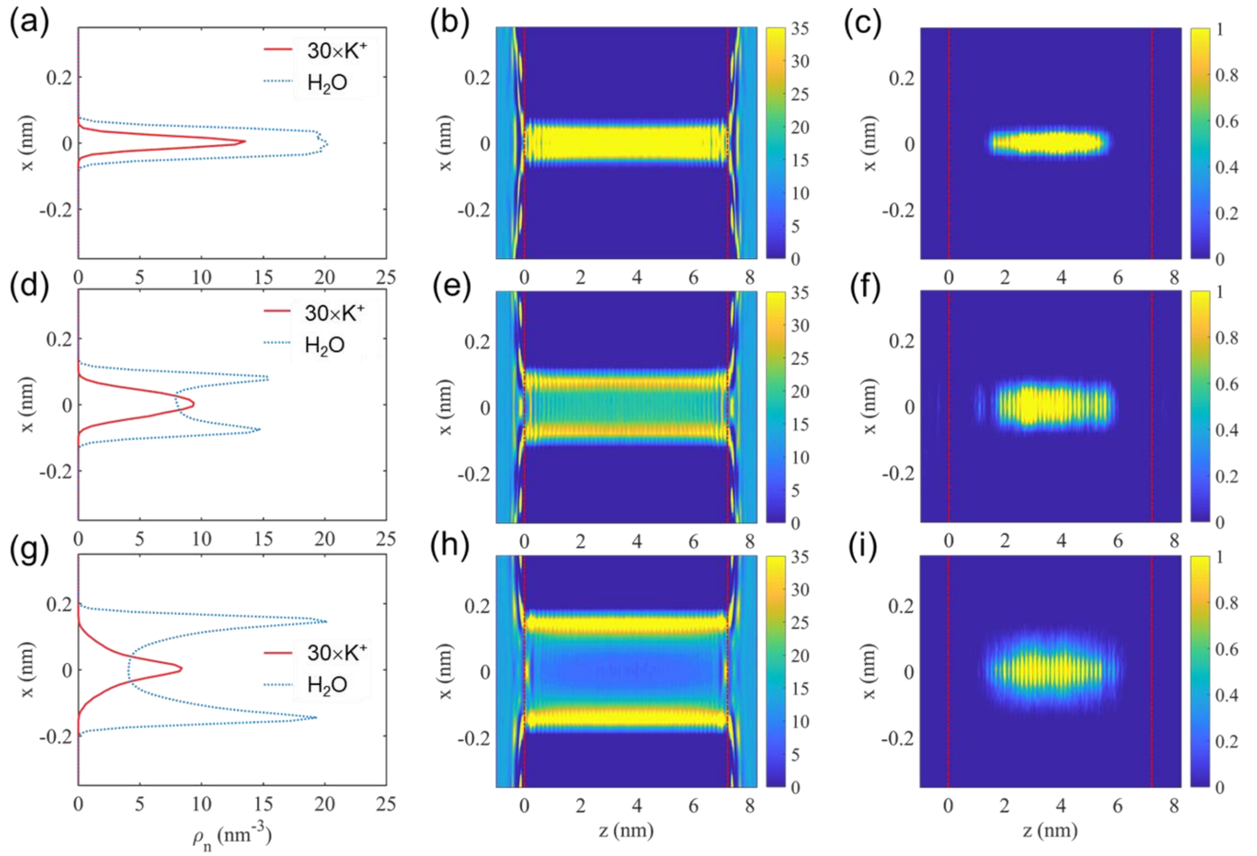

Supplementary Fig. S16. Number density distributions of cations and water molecules inside MXene nanochannels. (a,d,g) One-dimensional number density profiles of K^+ ions and water molecules along the confinement direction normal to the MXene plane (x-direction) for nanochannels with interlayer spacings of 2.7 Å (first row, **a**), 3.7 Å (second row, **d**), and 5.0 Å (bottom row, **g**). The x-direction is defined as the direction normal to the MXene basal plane (confinement direction). (**b,e,h**) Two-dimensional number density maps of water molecules in the x–z plane for MXene nanochannels with interlayer spacings of 2.7 Å (**b**), 3.7 Å (**e**), and 5.0 Å (**h**). (**c,f,i**) Two-dimensional number density maps of K^+ ions in the x–z plane for MXene nanochannels with interlayer spacings of 2.7 Å (**c**), 3.7 Å (**f**), and 5.0 Å (**i**).

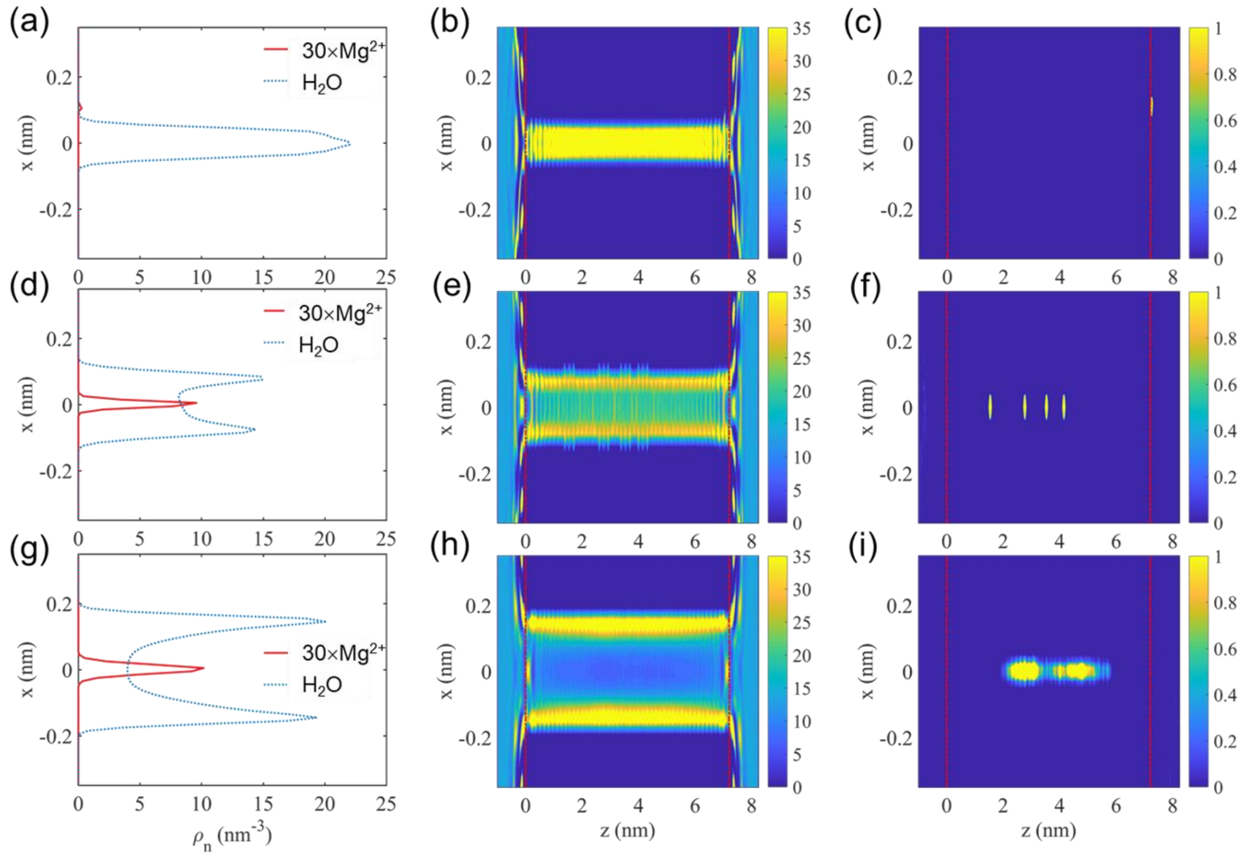

Supplementary Fig. S17. Number density distributions of cations and water molecules inside MXene nanochannels. (a,d,g) One-dimensional number density profiles of Mg^{2+} ions and water molecules along the confinement direction normal to the MXene plane (x-direction) for nanochannels with interlayer spacings of 2.7 Å (first row, **a**), 3.7 Å (second row, **d**), and 5.0 Å (bottom row, **g**). The x-direction is defined as the direction normal to the MXene basal plane (confinement direction). (**b,e,h**) Two-dimensional number density maps of water molecules in the x–z plane for MXene nanochannels with interlayer spacings of 2.7 Å (**b**), 3.7 Å (**e**), and 5.0 Å (**h**). (**c,f,i**) Two-dimensional number density maps of Mg^{2+} ions in the x–z plane for MXene nanochannels with interlayer spacings of 2.7 Å (**c**), 3.7 Å (**f**), and 5.0 Å (**i**).

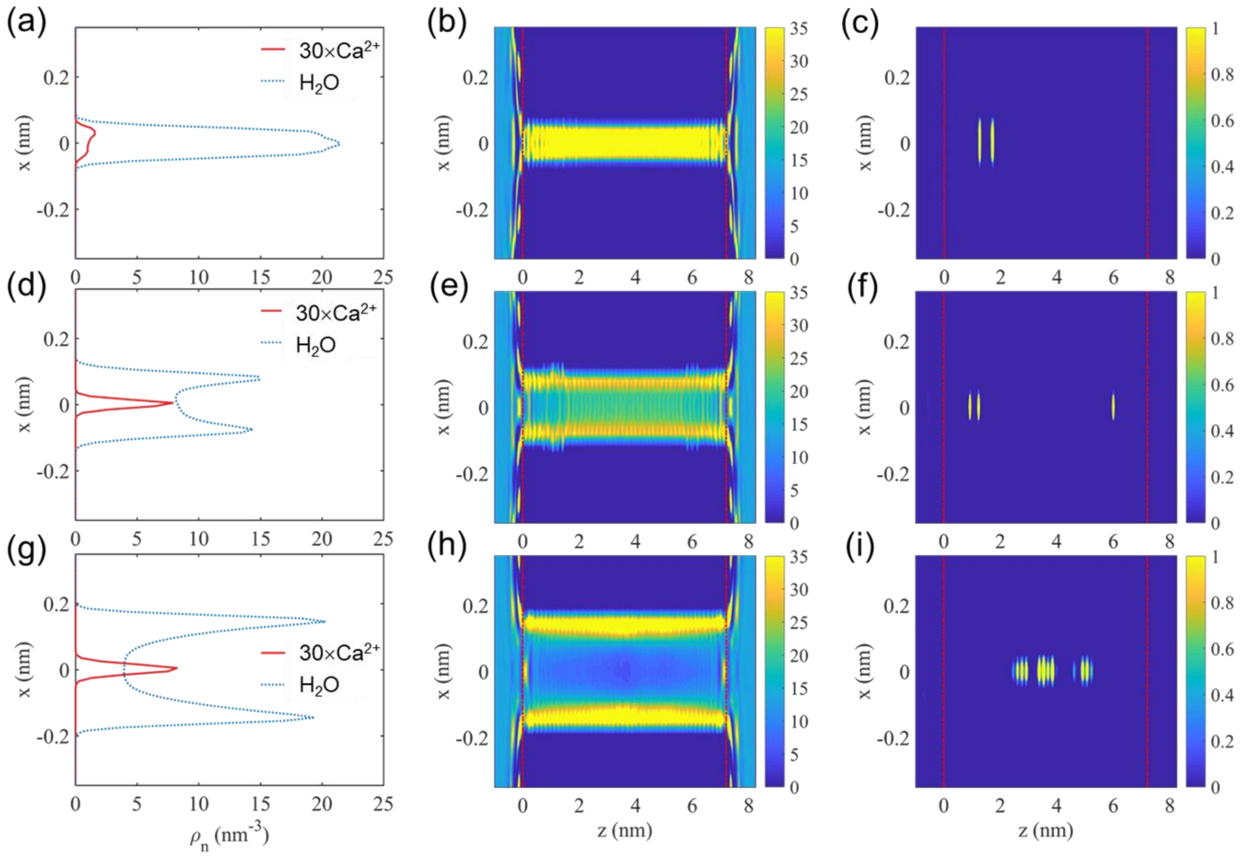

Supplementary Fig. S18. Number density distributions of cations and water molecules inside MXene nanochannels. (a,d,g) One-dimensional number density profiles of Ca^{2+} ions and water molecules along the confinement direction normal to the MXene plane (x-direction) for nanochannels with interlayer spacings of 2.7 Å (first row, **a**), 3.7 Å (second row, **d**), and 5.0 Å (bottom row, **g**). The x-direction is defined as the direction normal to the MXene basal plane (confinement direction). (**b,e,h**) Two-dimensional number density maps of water molecules in the x–z plane for MXene nanochannels with interlayer spacings of 2.7 Å (**b**), 3.7 Å (**e**), and 5.0 Å (**h**). (**c,f,i**) Two-dimensional number density maps of Ca^{2+} ions in the x–z plane for MXene nanochannels with interlayer spacings of 2.7 Å (**c**), 3.7 Å (**f**), and 5.0 Å (**i**).

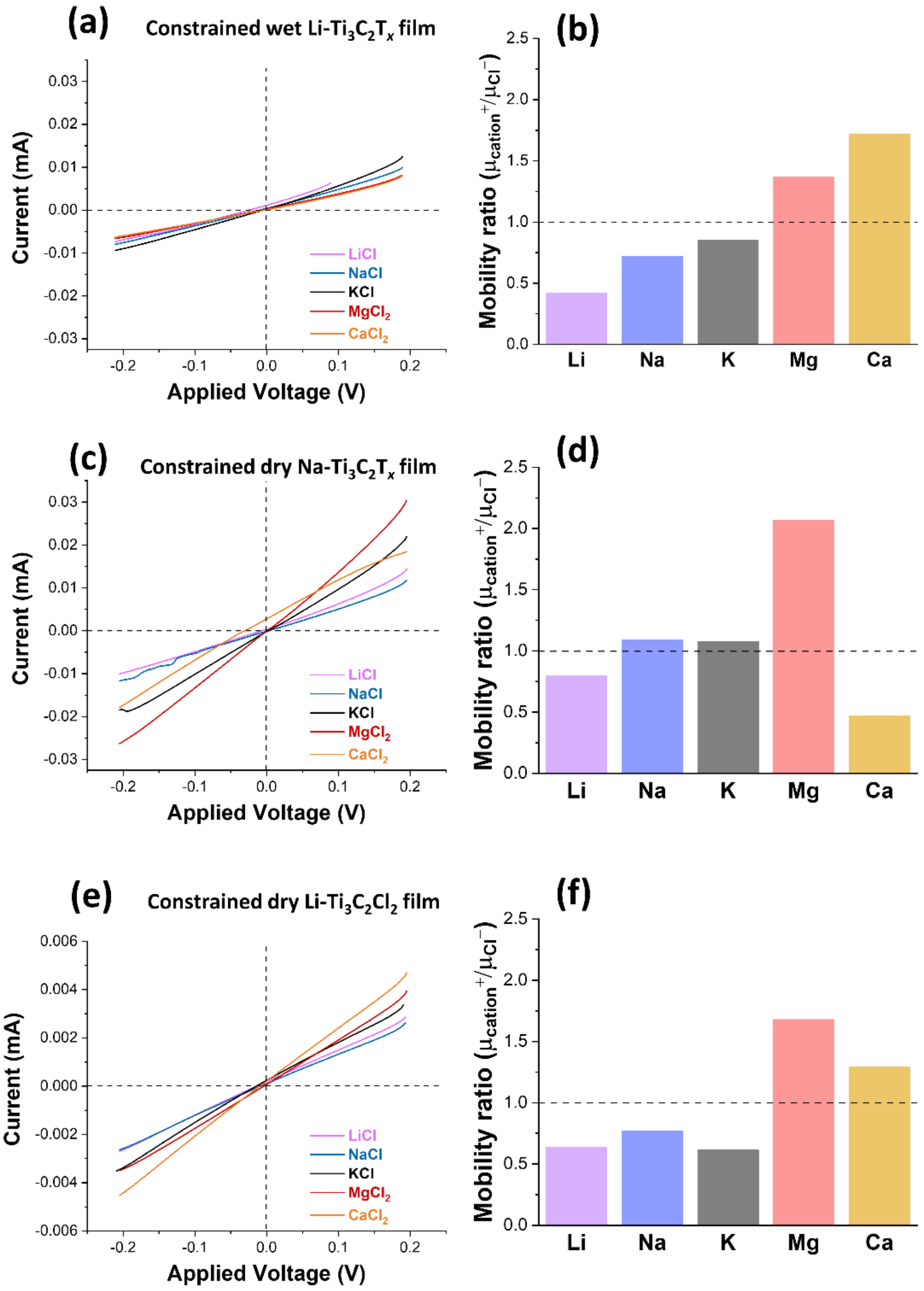

Supplementary Fig. S19. Drift diffusion measurements. (a-b) Constrained wet Li-$Ti_3C_2T_x$ channel. (**a**) *I-V* curves; (**b**) calculated mobility ratio of cations to anions. (c-d) Constrained dry Na-$Ti_3C_2T_x$ channel. (**c**) *I-V* curves; (**d**) calculated mobility ratio of cations to anions. (e-f) Constrained dry Li-$Ti_3C_2Cl_2$ channel. (**e**) *I-V* curves; (**f**) calculated mobility ratio of cations to anions.

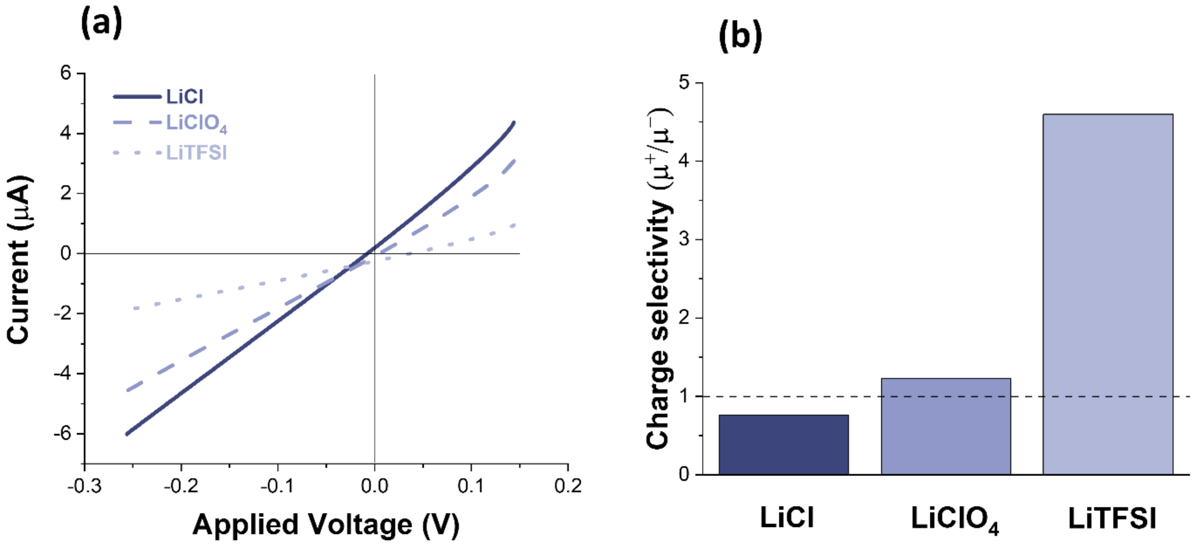

Supplementary Fig. S20. Drift diffusion measurement of Lithium salts with different anions in Li-$Ti_3C_2T_x$ channel. (**a**) *I*-*V* curves; (**b**) calculated charge selectivity of cations to anions.

Supplementary Table 1. Bare and hydrated ion sizes with different hydration energies [17-19].

Ion	Bare ion diameter (Å)	Hydration energy (ΔG^0_{hyd} /kJ mol^{-1})	Hydrated ion size (Å)
Li^+	1.38	-475	8.23
Na^+	2.04	-365	7.16
K^+	2.76	-295	6.62
Mg^{2+}	1.24	-1830	8.56
Ca^{2+}	1.98	-1505	8.24
Cl^-	3.62	-340	6.64

Supplementary Table 2. Atomic Li:Ti ratio of Li-$Ti_3C_2T_x$ and Li-$Ti_3C_2Cl_2$ from ICP-OES measurement.

Li-$Ti_3C_2T_x$	0.14: 1
Li-$Ti_3C_2Cl_2$	0.11:1

References

1 Han, M. *et al.* Electrochemically modulated interaction of MXenes with microwaves. *Nat Nanotechnol* **18**, 373-379 (2023).

2 Li, M. *et al.* Element Replacement Approach by Reaction with Lewis Acidic Molten Salts to Synthesize Nanolaminated MAX Phases and MXenes. *J. Am. Chem. Soc.* **141**, 4730-4737 (2019).

3 Ding, H. *et al.* Chemical scissor-mediated structural editing of layered transition metal carbides. *Science* **379**, 1130-1135 (2023).

4 Zhang, T. *et al.* Delamination of Chlorine-Terminated MXene Produced Using Molten Salt Etching. *Chem. Mater.* **36**, 1998-2006 (2024).

5 Michałowski, P. P. Secondary ion mass spectrometry measurements with a large scale-to-resolution ratio. *Appl. Surf. Sci.* **702**, 163272 (2025).

6 Parker, T. *et al.* Fourier-Transform Infrared Spectral Library of MXenes. *Chem. Mater.* **36**, 8437-8446 (2024).

7 Hess, B., Kutzner, C., van der Spoel, D. & Lindahl, E. GROMACS 4: Algorithms for Highly Efficient, Load-Balanced, and Scalable Molecular Simulation. *J. Chem. Theory Comput.* **4**, 435-447 (2008).

8 Berendsen, H. J. C., Grigera, J. R. & Straatsma, T. P. The missing term in effective pair potentials. *J. Phys. Chem.* **91**, 6269-6271 (1987).

9 Dezfoli, A. A., Mehrabian, M. A. & Hashemipour, H. Molecular Dynamics Simulation of Heavy Metal Ions in Aqueous Solution Using Lennard-Jones 12-6 Potential. *Chem. Eng. Commun.* **202**, 1685-1692 (2015).

10 Bussi, G., Donadio, D. & Parrinello, M. Canonical sampling through velocity rescaling. *J. Chem. Phys.* **126**, 014101 (2007).

11 Gingrich, T. R. & Wilson, M. On the Ewald summation of Gaussian charges for the simulation of metallic surfaces. *Chem. Phys. Lett.* **500**, 178-183 (2010).
12 Bi, S. *et al.* Molecular understanding of charge storage and charging dynamics in supercapacitors with MOF electrodes and ionic liquid electrolytes. *Nat. Mater.* **19**, 552-558 (2020).
13 Zeng, L. *et al.* Modeling galvanostatic charge–discharge of nanoporous supercapacitors. *Nat. Comput. Sci.* **1**, 725-731 (2021).
14 Chen, Y., Huang, Q., Liu, T.-H., Qian, X. & Yang, R. Effect of solvation shell structure on thermopower of liquid redox pairs. *EcoMat.* **5**, e12385 (2023).
15 Allen, T. W., Andersen, O. S. & Roux, B. Molecular dynamics — potential of mean force calculations as a tool for understanding ion permeation and selectivity in narrow channels. *Biophys. Chem.* **124**, 251-267 (2006).
16 Kumar, S., Rosenberg, J. M., Bouzida, D., Swendsen, R. H. & Kollman, P. A. THE weighted histogram analysis method for free-energy calculations on biomolecules. I. The method. *J. Comput. Chem.* **13**, 1011-1021 (1992).
17 Kubota, S., Ozaki, S., Onishi, J., Kano, K. & Shirai, O. Selectivity on Ion Transport across Bilayer Lipid Membranes in the Presence of Gramicidin A. *Analytical Sciences* **25**, 189-193 (2009).
18 Marcus, Y., Thermodynamics of solvation of ions. Part 5.—Gibbs free energy of hydration at 298.15 K. *J. Chem. Soc., Faraday Trans.* **87**, 2995–2999 (1991).
19 Nightingale, E. R. Phenomenological Theory of Ion Solvation. Effective Radii of Hydrated Ions. *The Journal of Physical Chemistry* **63**, 1381-1387 (1959).
